# Study design requirements for RNA sequencing-based breast cancer diagnostics

**DOI:** 10.1038/srep20200

**Published:** 2016-02-01

**Authors:** Arvind Singh Mer, Daniel Klevebring, Henrik Grönberg, Mattias Rantalainen

**Affiliations:** 1 Department of Medical Epidemiology and Biostatistics, Karolinska Institute, Nobels Väg 12A, SE-17177, Stockholm, Sweden

## Abstract

Sequencing-based molecular characterization of tumors provides information required for individualized cancer treatment. There are well-defined molecular subtypes of breast cancer that provide improved prognostication compared to routine biomarkers. However, molecular subtyping is not yet implemented in routine breast cancer care. Clinical translation is dependent on subtype prediction models providing high sensitivity and specificity. In this study we evaluate sample size and RNA-sequencing read requirements for breast cancer subtyping to facilitate rational design of translational studies. We applied subsampling to ascertain the effect of training sample size and the number of RNA sequencing reads on classification accuracy of molecular subtype and routine biomarker prediction models (unsupervised and supervised). Subtype classification accuracy improved with increasing sample size up to N = 750 (accuracy = 0.93), although with a modest improvement beyond N = 350 (accuracy = 0.92). Prediction of routine biomarkers achieved accuracy of 0.94 (ER) and 0.92 (Her2) at N = 200. Subtype classification improved with RNA-sequencing library size up to 5 million reads. Development of molecular subtyping models for cancer diagnostics requires well-designed studies. Sample size and the number of RNA sequencing reads directly influence accuracy of molecular subtyping. Results in this study provide key information for rational design of translational studies aiming to bring sequencing-based diagnostics to the clinic.

Breast cancer is the second most common cancer worldwide and a leading cause of cancer-related mortality in women. It has been estimated that in 2012 alone there were 1.67 million new cases and 522,000 deaths attributed to breast cancer^1^. Histopathological features of the tumour play a central role in treatment decision making. Immunohistochemistry (IHC) and fluorescence *in situ* hybridisation (FISH) based diagnosis of pathological criteria such as receptor (HER2, ER, PR) status and proliferation (Ki67) are routinely used when deciding therapeutic procedure and treatment planning. Over the past decade, genetic analysis has revealed a detailed molecular portrait of breast cancer and established it as a heterogeneous disease^2^,^3^. Using clustering analyses of microarray based gene expression data, Perou *et al.*^4^ initially identified four breast cancer intrinsic subtypes (basal-like, HER2-enriched, luminal and normal breast-like). Subsequent studies by Sorlie and colleagues^5^ led to sub-stratification of luminal breast cancers into luminal A (LumA) and luminal B (LumB). Several other studies confirmed the five intrinsic subtypes^6^,^7^ and established a working model for breast cancer molecular taxonomy. The classification system based on gene-expression data is known to provide improved prognosis over routine pathology^8^,^9^,^10^,^11^,^12^. Their clinical utility has been demonstrated with respect to personalized treatment in chemotherapy and hormonal therapy^13^,^14^,^15^,^16^. Given the advancement in sequencing technology we expect that the sequencing of tumours will become routine in clinical diagnosis and prognosis^17^,^18^,^19^.

Gene expression-based molecular subtyping has been applied to characterise tumors in multitude of cancer types, including prostate cancer^20^,^21^, ovarian cancer^22^, lung cancer^23^,^24^, colorectal cancer^25^, renal cancer^26^ and breast cancer^4^,^5^. Subtype-based classification has been demonstrated to provide improved patient stratification^27^ and has also been utilised to propose possible therapeutic interventions^28^,^29^,^30^. The establishment of subtype prediction models with high sensitivity and specificity is a key prerequisite for the adaptation of RNA sequencing-based subtyping of tumours in the clinical setting. Although RNA sequencing is known to have high reproducibility within a single laboratory and experimental protocol, the inter-lab and inter-protocol variability can be substantial^31^. Therefore, direct application of fitted models across laboratories may prove to be challenging. This challenge is likely to drive the generation of local in-house data sets to train and evaluate subtype prediction models during clinical translation. For routine clinical marker prediction, the effect of training sample size has previously been analysed in the context of microarray-based gene expression profiling in relatively small dataset (N = 230)^32^. However, so far there have not been any comprehensive studies that assess sample size requirements for molecular subtyping of breast cancer using RNAseq-based gene expression profiling and also considering larger datasets.

In this study we investigate the dependency of prediction accuracy on the training sample size (learning curve) using The Cancer Genome Atlas (TCGA) Data set^33^. In particular, we assess the sample size requirement for supervised and unsupervised molecular subtyping of breast cancer based on RNA sequencing. We investigate sample size requirements for classification models to determine the status of the routine markers ER, PR and HER2. We also investigate to what extent the number of RNAseq reads count influence prediction performance. Our results are directly relevant for anyone designing translational studies focused on patient stratification by molecular subtyping in breast cancer, and may also be relevant for study design in other cancers.

## Results

### Effect of sample size on subtype prediction

**Figure 1:**
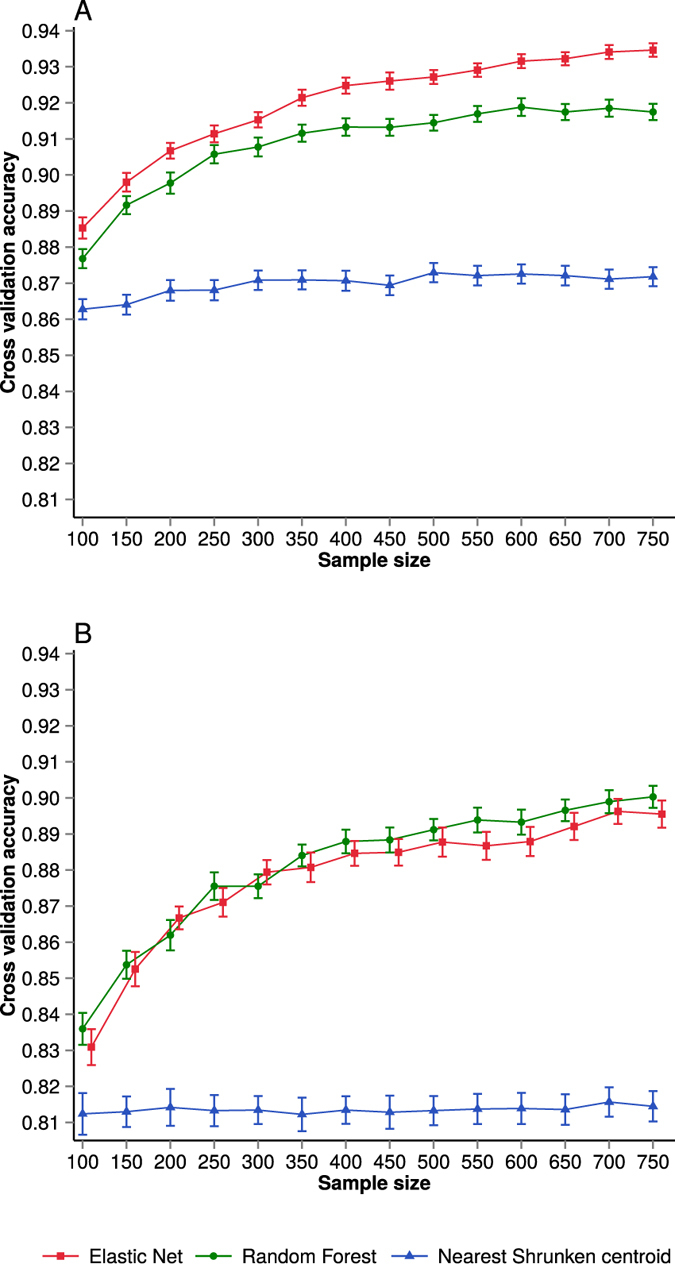
Subtype classification accuracy (nested cross-validation) as a function of training dataset sample size. Subtype classification accuracy (**A**) for the PAM50 data set and (**B**) for the Top25 dataset. Performances of different classifiers are indicated by distinct colours. For the purpose of visualisation, Elastic net data points in (**B**) have been shifted slightly to the right. Error bars represent standard error of the cross-validation mean.

To assess the effect of sample size on subtype classification, we examined how the performance of three different classification models (Elastic net, Random forest and Nearest shrunken centroids) varies as a function of the training sample size. From the TCGA Breast cancer data we created two datasets: PAM50 dataset, consist of only PAM50 features and Top25 dataset consist of top 25% features of highest variation. Sample size effect was evaluated for both datasets. Figure 1 shows 100 fold cross-validation average accuracy for each of the classifiers and datasets as the training sample size increased from 100 to 750 samples (see Supplementary figure S5 and S6 for visualisation of cross-validation test set accuracy variability over cross-validation rounds).

We observed a gain in performance of classifiers as the training sample size increases. However, the improvement in performance depends on the chosen classification model and also the dataset. The performance of Elastic net and Random forest classifiers improved with increasing training sample size, which is typically expected in classification problems with noisy data and not perfect class separation (Fig. 1). However, in the case of the Nearest shrunken centroid model, the gain in performance is small as sample size is increasing and the classification performance was poorer compared to the Elastic net and Random forest models. For the PAM50 dataset, the regularisation based classification method (Elastic net) provided marginally higher classification performance than did the Random forest model. In the analysis of the Top25 dataset, the difference in performance of Elastic net and the ensemble classification method (Random forest) was minimal (Fig. 1).

When we investigated the performance gain across both datasets (PAM50 and Top25), we observed that the dataset with a large number of features (Top25) made a relatively larger increase in classification performance as a function of increasing sample size compared to the small PAM50 dataset. A likely explanation of this difference is the fact that the PAM50 feature is a carefully selected set of genes that are informative for breast cancer subtype classification. We also note that the subtype labels used for benchmarking are based on the PAM50 gene panel.

For both regularisation and ensemble based classification, classification accuracy increases more rapidly as a function of the training sample size up to around N = 350. Beyond N = 350 observations, the increase in classification performance is more modest as learning curves start achieving a plateau.

**Figure 2:**
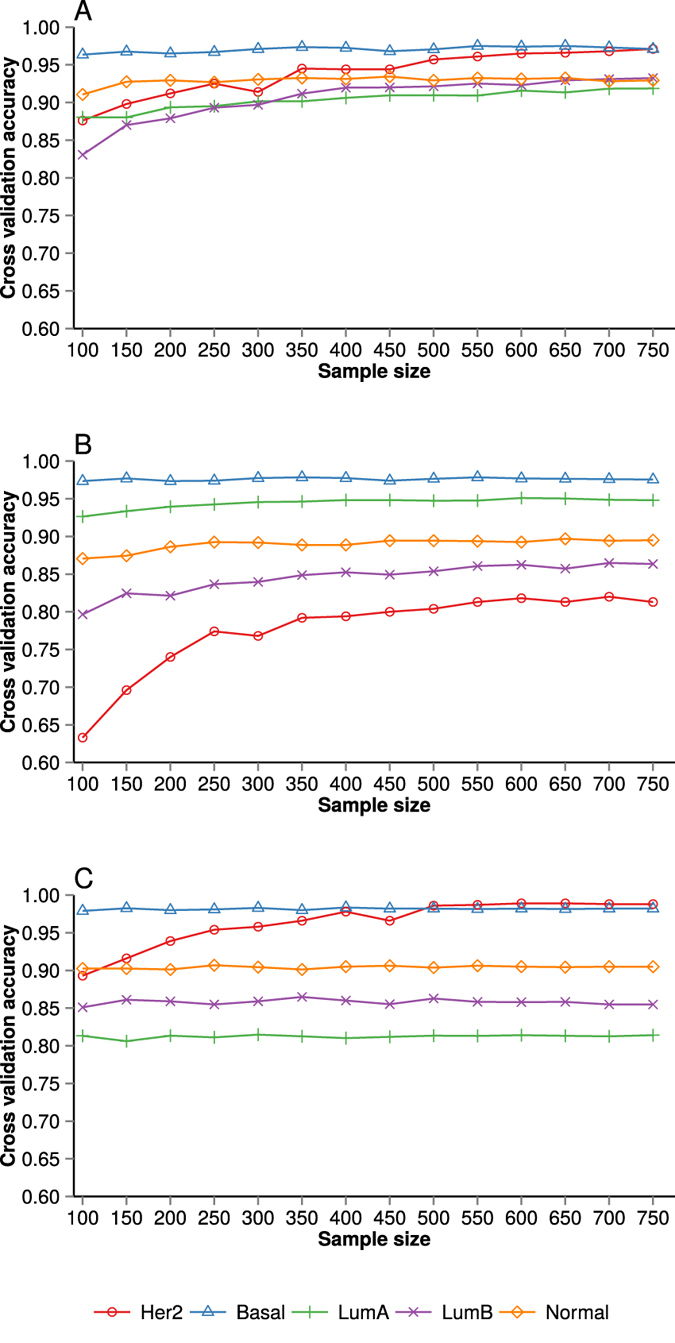
Subtype classification accuracy (nested cross-validation) as a function of training dataset sample size stratified by molecular subtype in the PAM50 dataset. (**A**) Elastic net (**B**) Random forest and (**C**) Nearest Shrunken centroid classifier. Error bars represent standard error of the cross-validation mean.

Figure 2 shows the cross-validation accuracy for individual subtypes at different training sample sizes. The results indicate that the performance of the Her2 subtype improves with an increase in training samples. The learning curve for the Basal and Normal subtypes achieves a plateau at smaller sample sizes. This observation is true for all three classifiers. A likely cause of the flat learning curve for Basal and Normal subtype is the fact that the histological and molecular properties of these subtypes are substantially different from other subtypes (Supplementary figure S1 and figure S2). This facilitates the recognition and classification of the Basal subtype even at low training sample size. On the contrary, LumA and LumB subtypes are very similar at molecular level, sharing the property of both being ER positive, therefore a larger training sample size is required to improve classification performance for LumA and LumB. However, irrespective of larger training sample sizes, some samples from these two groups are likely to remain hard to classify consistently, suggesting a potential continuum in molecular differences between LumA and LumB subtypes. Confusion matrix for Top25 dataset using Elastic net classifier at sample size of N = 750 is shown in Table 1. Detailed confusion matrix (using Elastic net classifier) for PAM50 and Top25 dataset can be found in Supplementary Table S1 and Table S2 respectively.

### Sample size and receptor status

**Figure 3:**
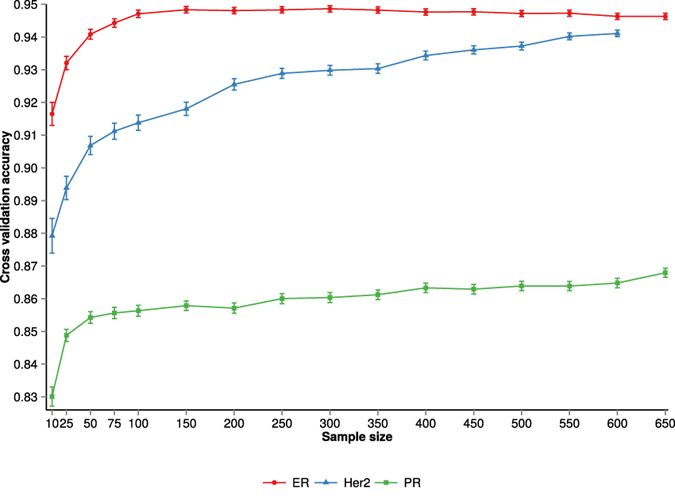
Effect of training dataset sample size on receptor status prediction performance. The cross-validation accuracy and standard error of the mean for ER, PR and Her2 receptor status is plotted against the training sample size.

To assess the effect of sample size on receptor status prediction from RNASeq data we examined ER, PR and Her2 receptor status classification performance. Figure 3 shows the cross-validation accuracy and standard error of the cross-validation mean for ER, PR and Her2 receptor status classification. The ER and Her2 models achieve high accuracy at even relatively small sample sizes, with accuracies >0.94 at N = 200 for ER status and >0.92 for Her2 status classification. Prediction performance for PR (<0.86) at N = 200 is lower than that of ER and Her2. The prediction accuracy for Her2 status improves gradually with increasing sample size, in contrast to the ER model that is saturated already at N = 150 observations.

### Read count per sample affects classifier performance

**Figure 4:**
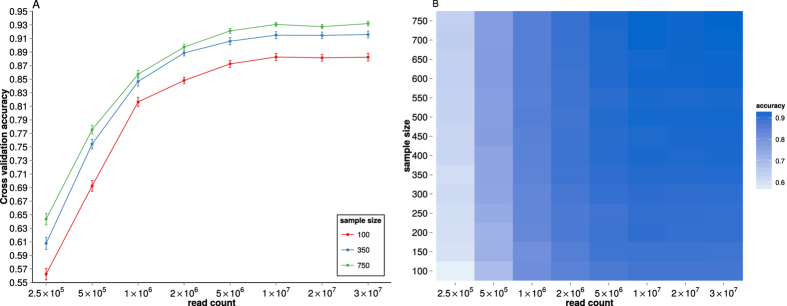
Effect of read count per sample on subtype prediction in PAM50 dataset. (**A**) Classification accuracy at training sample size of 100, 350 and 750 (Error bars represent standard error of the cross-validation mean). (**B**) Heatmap representation of subtype classification accuracy and read count per sample.

Next we assessed the effect of read count per sample on classification performance by subsampling the RNAseq reads and estimating the classification performance in respect to subtype. Figure 4 shows how the classification accuracy is affected by the RNAseq read count for different sample sizes. We note that, in case of PAM50 dataset, at 5 × 10^6^ reads per sample, the increase in classification accuracy is not improving, suggesting that subtype classification can be accomplished at this read count. It is important to note that the read counts referred to here are read counts that are mapped correctly and annotated as protein coding. For the Top25 dataset, the increase in classification accuracy starts to stagnate at 2 × 10^6^ reads per sample (supplementary figure S7) but at slightly lower accuracy then PAM50 dataset.

### Effect of sample size on unsupervised learning

**Figure 5:**
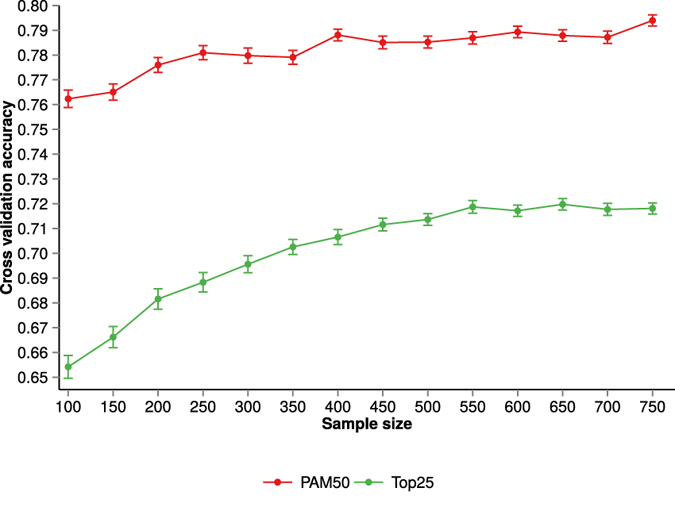
Subtype classification accuracy (nested cross-validation) as a function of training dataset sample sizes in the case of unsupervised classification case. Results are based on 100-fold cross-validation using the PAM50 dataset and the Top25 dataset. Error bars represent standard error of the cross-validation mean.

Training sample size has a clear effect on performance of unsupervised classification (Fig. 5). As expected, we found that the unsupervised classification performance is consistently lower than the supervised case (see previous sections). The lower classification accuracy of the cross-validated unsupervised model can, however, to a large extent be attributed to misclassification between LumA and LumB subtypes, which are expected to be fairly similar (confusion matrices can be found in supplementary table S3 and S4).

## Discussion

Molecular characterisation of tumours is a primary building block in the process of enabling personalised cancer diagnostics and treatment. DNA and RNA sequencing technology is now mature and ready to be translated to the clinic to improve prognostication and patient stratification.

To provide consistent molecular subtyping of tumours, multivariate prediction models have to be implemented that take the molecular profile of the tumour as input and return subtype labels and associated probabilities. The training of prediction models, including optimisation of potential tuning parameters and estimation of model parameters, requires data to learn from. With increasing sample size of the training data, one can generally expect improvement of the prediction performance towards some upper limit. The upper limit will be determined by noise present in the training data labels, e.g. inconsistency or uncertainty in clinical assignments of ER and Her2 status, or noise (biological and technical) in the molecular profiling data. In the case of “easy” classification problems, where there are large molecular differences between the groups, smaller training datasets are required to estimate a good model, while in more challenging classification problems, i.e. with smaller effect sizes and more subtle differences between groups, larger training data sets are required. In biomedical applications the study sample size is usually limited by either economical considerations or by the availability of biological material, e.g. tumour samples, biopsies or other biological samples, or by both. Study design is therefore a key consideration to ensure studies with sufficient sample sizes to produce the required results, which in this case would be defined by models that can be used in the clinical context.

Previous studies of sample size effects on classification models with molecular predictors were based on microarray-based gene expression profiling data and smaller sample sizes^32^, or focused on other related classification problems, including network-based classifiers^34^ and sample size effects on feature selection^35^. In this study our aim was to estimate the influence of the training set sample size on the accuracy in the prediction of routine biomarker status from RNAseq data and the accuracy in prediction of molecular subtypes in breast cancer. In particular we assessed how sample size and read depth of the training data set influence statistical power in the predictive sense, i.e. the learning curve, for models based on gene expression profiling by RNAseq. The results have direct implication for those who plan to translate sequencing-based molecular profiling of breast cancer tumours to the clinic in order to provide improved prognostication and patient stratification.

In the case of breast cancer, we found that the prediction accuracy is improving as a function of sample size for both molecular subtyping as well as for prediction of routine markers throughout the sample size range we have investigated (N = 100 to N = 750). However, the improvement is more dramatic up to around N = 350 in the case of subtyping, after which the rate of improvement in accuracy is slowing down. i.e. the cost of improving e.g. 1% in accuracy is gradually increasing as the sample size gets larger.

The unsupervised molecular subtype problem is a harder one than supervised learning. This was indicated by the substantially lower classification performance in the unsupervised case. A large part of the misclassification can, however, be attributed to samples within the luminal group that gets misclassified between the LumA and LumB groups.

It is important to realise that the quality of the clinical data also plays an important role for the classification accuracy that can be achieved. In retrospective material and biobanks, there is almost always a degree of mislabelling, errors in clinical meta data and other issues that will put an upper bound on how good models can be developed.

We also investigated the impact of RNAseq read depth, or rather the number of RNAseq reads, on prediction accuracy. For subtype prediction we found that the prediction performance did not improve as the number of mapped reads of protein coding transcripts reached 5 million. However in the case of the Top25 dataset prediction performance stagnates at about 2 million reads. It is important to realise that this figure is only valid for the particular challenge of predicting the intrinsic subtype, and not for other scientific questions that might be put forward. We also expect that the number of reads that are required will increase as the number of subtypes gets larger in alternative subtype models^36^.

Sequencing-based molecular characterisation of breast cancer tumours is expected to provide direct patient benefits due to a more precise characterisation of the molecular aetiology of the tumour and will lead to improved prognostication, patient stratification and ultimately a more personalised treatment. The results presented here provide guidance for study design of translational breast cancer genomics studies aiming to implement models for prediction of routine markers and for molecular subtyping.

## Methods

### Dataset

In this study we utilize publicly available TCGA RNAseq and clinical data, in accordance with the TCGA data access and publication guidelines. For all samples materials and clinical data, informed consent and ethics approval have obtained by TCGA, based on guidelines laid out by the TCGA Ethics, Law and Policy Group. Clinical data from the TCGA invasive breast carcinoma dataset (provisional) was downloaded from the TCGA data portal (https://tcga-data.nci.nih.gov/tcga/) on 11th of December 2013 and included data for 1148 cases. Unaligned RNAseq data from the TCGA dataset was downloaded (June 2014) after approval from the TCGA data access committee (N = 1126, all available cases with unaligned data). 1073 cases were available with both unaligned paired-end RNAseq data and clinical data. Out of these, 35 observations were excluded as potential outliers based on inspection of Principal Component Analysis scores and residuals. In the remaining data, 885 samples had molecular subtype (PAM50) assignments available and each has 20477 gene expression features. The dataset has five different subtypes: Basal (=132), Her2 (=65), LumA (=393), LumB (=190), Normal (=105). We calculated the variance for each feature and selected the top 25% features. This dataset, containing 885 samples and 5120 features, was called Top25 dataset. We created another dataset, including PAM50 features only and named it PAM50 dataset^37^.

### Bioinformatic pre-processing

Standard Illumina adapters (AGATCGGAAGAGCACACGTCTGAACTCCAGTCAC and AGATCGGAAGAGCGTCGTGTAGGGAAAGAGTGTA) were trimmed using skewer version-0.1.117^38^ with default parameters. Alignment was carried out using STAR aligner version-2.4.0e^39^ with the following parameters: “–outSAMmapqUnique 50”, to set the maximum alignment quality score to 50; “–outSAMunmapped Within”, to include unmapped reads in the resulting SAM file; “–chimSegmentMin 20” to require that a minimum of 20 bases maps to each end of a chimeric transcript (output in a separate file) and “–outSAMattributes NH HI AS nM NM MD XS” to include additional attributes in the SAM file. Gene expression estimates were calculated with HTSeq count version-0.6.0^40^ with the following parameters: “–stranded = no” and “–mode = intersection-nonempty” for counting reads using the default alignment quality filter threshold of 10. To evaluate the effect of a higher quality score threshold, we used the parameter “-a 30”. Data were normalised using the DESeq method^41^.

### Prediction modelling and estimation of subtype classification performance

We examined three different multivariate prediction models in combination with the previously mentioned datasets: Elastic net^42^, Random forest and Nearest shrunken centroid (NSC)^43^,^44^. The Elastic net is a regularised regression method that combines L1 (lasso) and L2 (ridge) penalties^42^. The Elastic net regularisation encourages a grouping effect which makes it particularly useful for analysis of genomic data, where the number of variables is much larger than the number of observations.

The Random forest method is based on an ensemble of decision trees. It constructs a large set of decision trees using bootstrap sampling and random subset of the variables. The outcome is predicted by averaging over the trees. During the training, trees are not pruned which creates trees with low-bias. The decision tree method is known to over-fit, while Random forest overcomes this problem by bagging^45^ and by applying a random variable selection approach. Moreover, the Random forest classifier does not require feature selection as it performs well with data of high dimension and with noisy predictors^46^.

The Nearest Shrunken Centroid method is a modification of the standard nearest centroid approach. NSC has been extensively used for microarray data analysis^43^,^44^. Similar to the standard nearest centroid approach, it uses the nearest centroid rule for the classification. However, in the Nearest shrunken centroid method, each class centroid is shrunken towards the overall centroid for all classes. The shrinkage of class centroids reduces the impact of noisy variables.

Given a training dataset of size *N*, for each prediction model the following parameters were optimised: for Elastic net alpha and lambda were optimised; for Random forest models the number of trees and the number of variables per level were optimised and for Nearest shrunken centroid models the parameter shrinkage threshold was optimised.

To estimate prediction performance we apply cross-validation. Since we have to optimise model parameters empirically while also estimating prediction performance, we applied a nested cross-validation approach. For a cross-validation cycle the procedure is as follows: In the first step, the test dataset which contains 10% original data is selected using a stratified, i.e. class-balanced, random sampling procedure. Thus, in the test dataset, subtype classes are in the same proportion as in the original dataset. This dataset is not used during any training and optimisation process and thus can be considered as an external test dataset in each “outer” cross-validation round. From the remaining dataset, we selected the training sets of different sample sizes ranging from 100 to 750 samples. This selection of different sample sizes training dataset is also carried out using stratified random sampling procedure. The training dataset is used in parameter optimisation for the prediction models, while the prediction performance is evaluated based on the “outer” test dataset and the optimised prediction model.

To assess effects of RNA read count on classification performance, reads were sub-sampled at different read count per sample and subsequently normalised. Performance of optimised prediction models was assessed using overall classification accuracy and class balanced classification accuracy in the multi-class case. Accuracy is defined as number of correctly predicted cases across all classes, divided by the number of total cases. Given the high degree of class imbalance in the dataset, the overall accuracy may lead to erroneous interpretation^47^. To overcome this problem we also computed the balanced accuracy which is defined as the average of all class-wise accuracy (see supplementary figure S3 and S4).

To assess the effect of training sample size on receptor status prediction, we analyse three clinicopathological relevant receptors, estrogen receptor (ER), progesterone receptor (PR) and Her2 receptor (Her2). The predictions were made using a logistic regression model with the clinical receptor status (positive/negative) as the response variable and using the expression level of ESR1 as the predictor in the ER model, expression of PGR as the predictor in the PR model and expression level of ERBB2 as the predictor in the Her2 model. For each cross-validation round 10% of the dataset is selected as test dataset using a stratified random sampling procedure. From the remaining data, training sets of different sample sizes were selected. The class probabilities were estimated for the training sets by applying logistic regression model and were used for calculating receiver operating characteristic (ROC) curve. An optimal cut-off point for receptor status was calculated from the ROC curve and Youden’s index method. Using the optimal cut-off point, labels for the test dataset were predicted and accuracy was calculated.

### Analysis using unsupervised learning

In the unsupervised setting we started with molecular profiling data with the aim of inferring distinct subtypes of cases through clustering. Here, we applied the approach originally proposed by Parker *et al.*^37^. The samples were analysed using hierarchical clustering using correlation-based distance and average linkage. The tree is pruned to obtain five significantly reproducible clusters by using the *SigClust* method^48^ with centroids calculated for each respective cluster. These centroids were used for classification of samples into different clusters, using the Nearest shrunken centroid classifier. For this analysis we utilised the implementation provided in the *Genefu* package^49^. The subtype label of each cluster was assigned by means of maximum Spearman’s rank correlation between each cluster centroid and the TCGA subtype centroids. The subtype labels were assigned to the clusters with the following priority order: Basal, Normal, Her2, LumA, LumB. The centroids and associated subtype labels were subsequently used for classification of observations in the test dataset and cross-validation classification accuracy was assessed.

## Additional Information

**How to cite this article**: Mer, A. S. *et al.* Study design requirements for RNA sequencing-based breast cancer diagnostics. *Sci. Rep.* **6**, 20200; doi: 10.1038/srep20200 (2016).

## Supplementary Material

Supplementary Information

## Figures and Tables

**Table 1:** Patterns of misclassification as described by the confusion matrix for sample size N = 750 in the Top25 dataset with classifications by the Elastic net model.

	Predicted label					
		Her2	Basal	LumA	LumB	Normal
True label	Her2	0.902	0.008	0.022	0.068	0
	Basal	0.038	0.961	0	0.001	0
	LumA	0.018	0	0.901	0.062	0.02
	LumB	0.031	0	0.137	0.832	0
	Normal	0.018	0.019	0.04	0.019	0.905
