# Supplementary material for: Study design requirements for RNA sequencing-based breast cancer diagnostics
Source: Sci Rep. 2016 Feb 1;6:20200. doi: 10.1038/srep20200 (PMC4735337; doi:10.1038/srep20200)
Supplement: Supplementary Information [file srep20200-s1.pdf]

Supplementary Information

Study design requirements for RNA sequencing-based breast cancer diagnostics

Arvind Singh Mer, Daniel Klevebring, Henrik Gronberg and Mattias Rantalainen,

Department of Medical Epidemiology and Biostatistics,

Karolinska Institute,

Nobels Väg 12A, SE-17177

Stockholm, Sweden

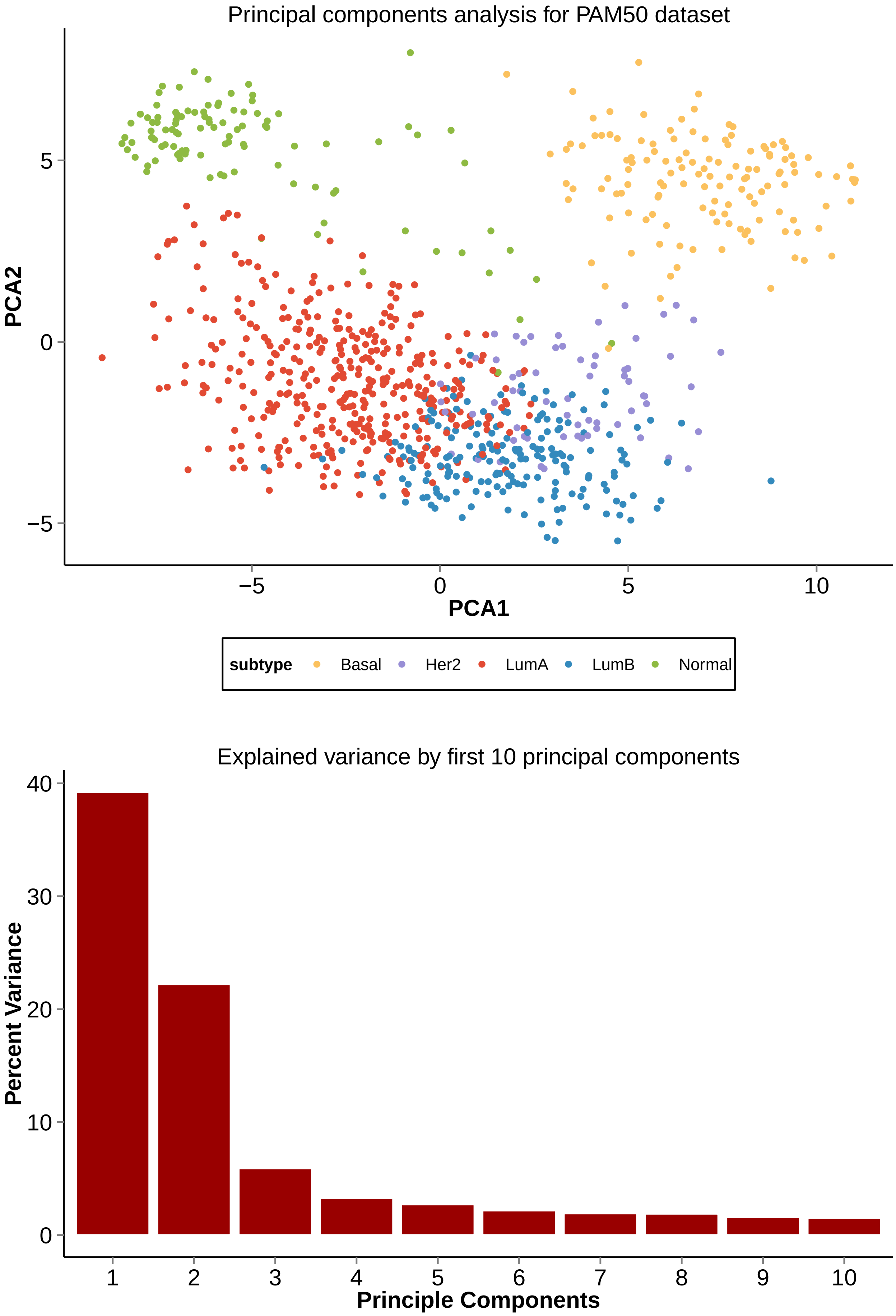

Figure S1: Principal component analysis of PAM50 dataset.

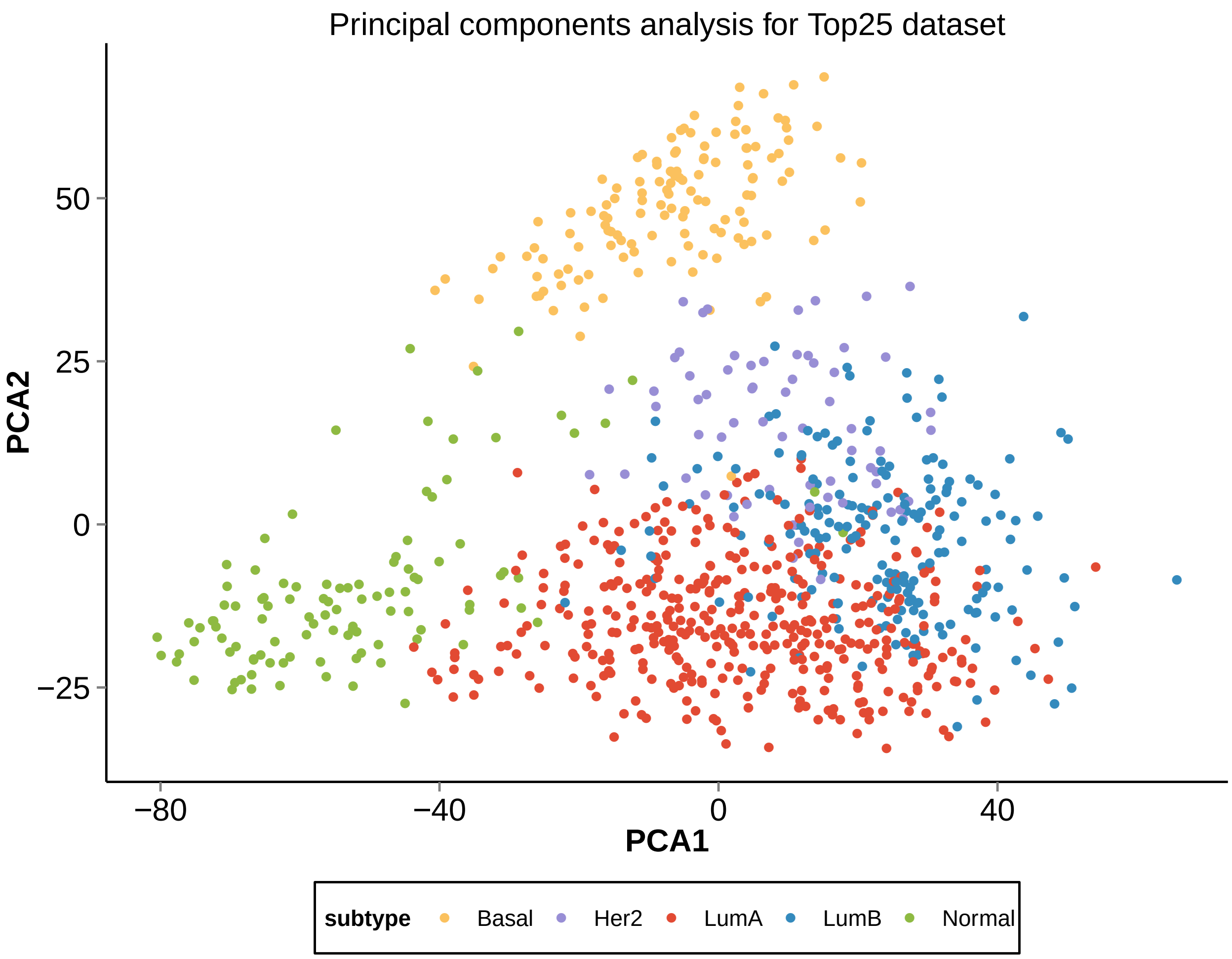

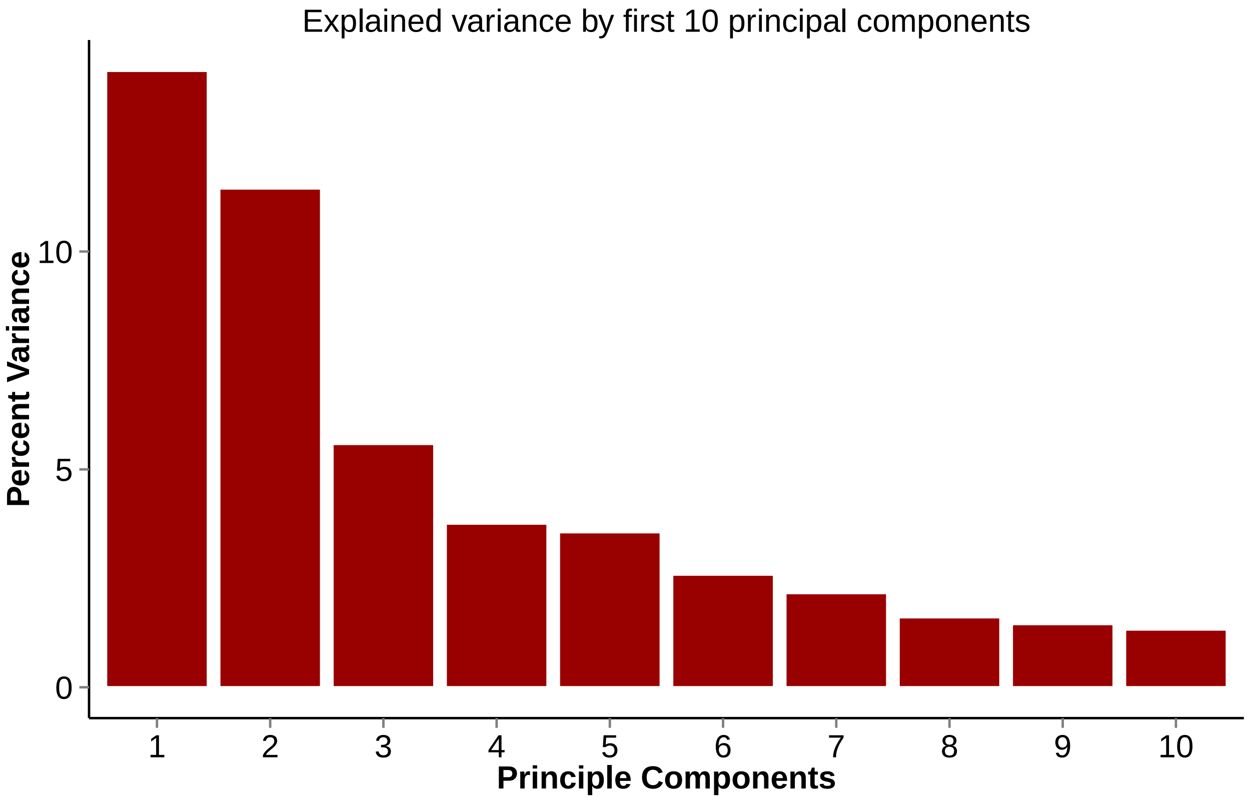

Figure S2: Principal component analysis of Top25 dataset.

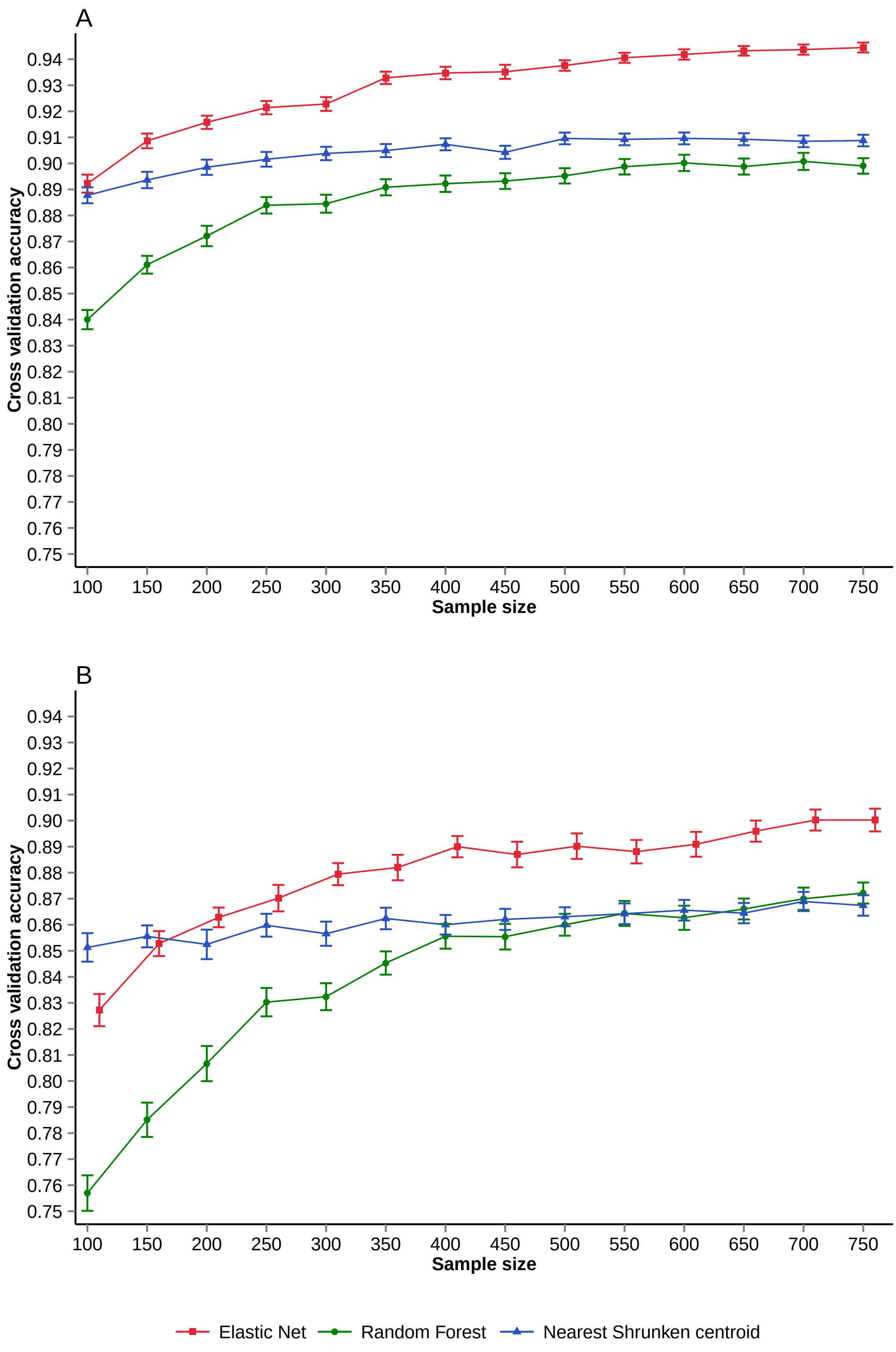

Figure S3: Balance accuracy for (A) PAM50 and (B) Top25 dataset.

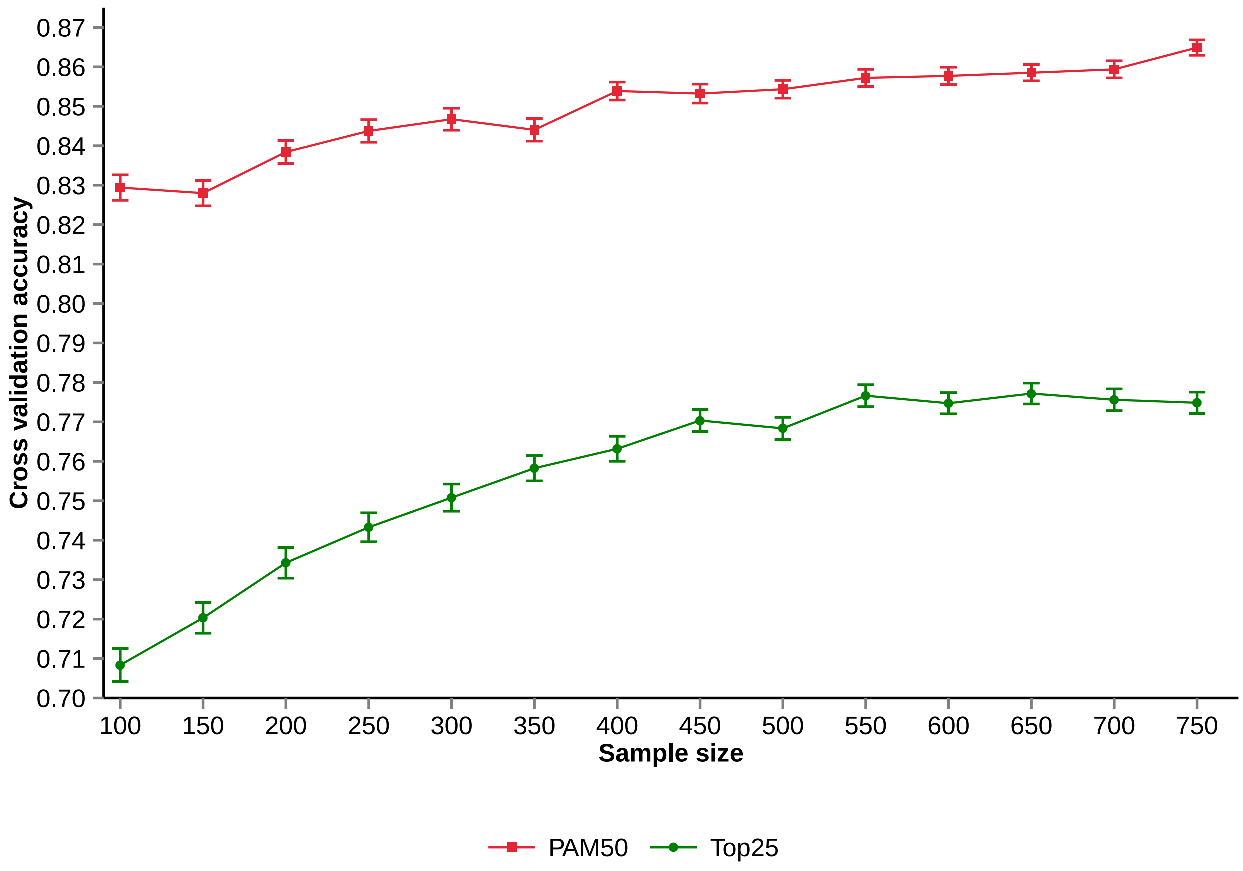

Figure S4: Balance accuracy for unsupervised classification.

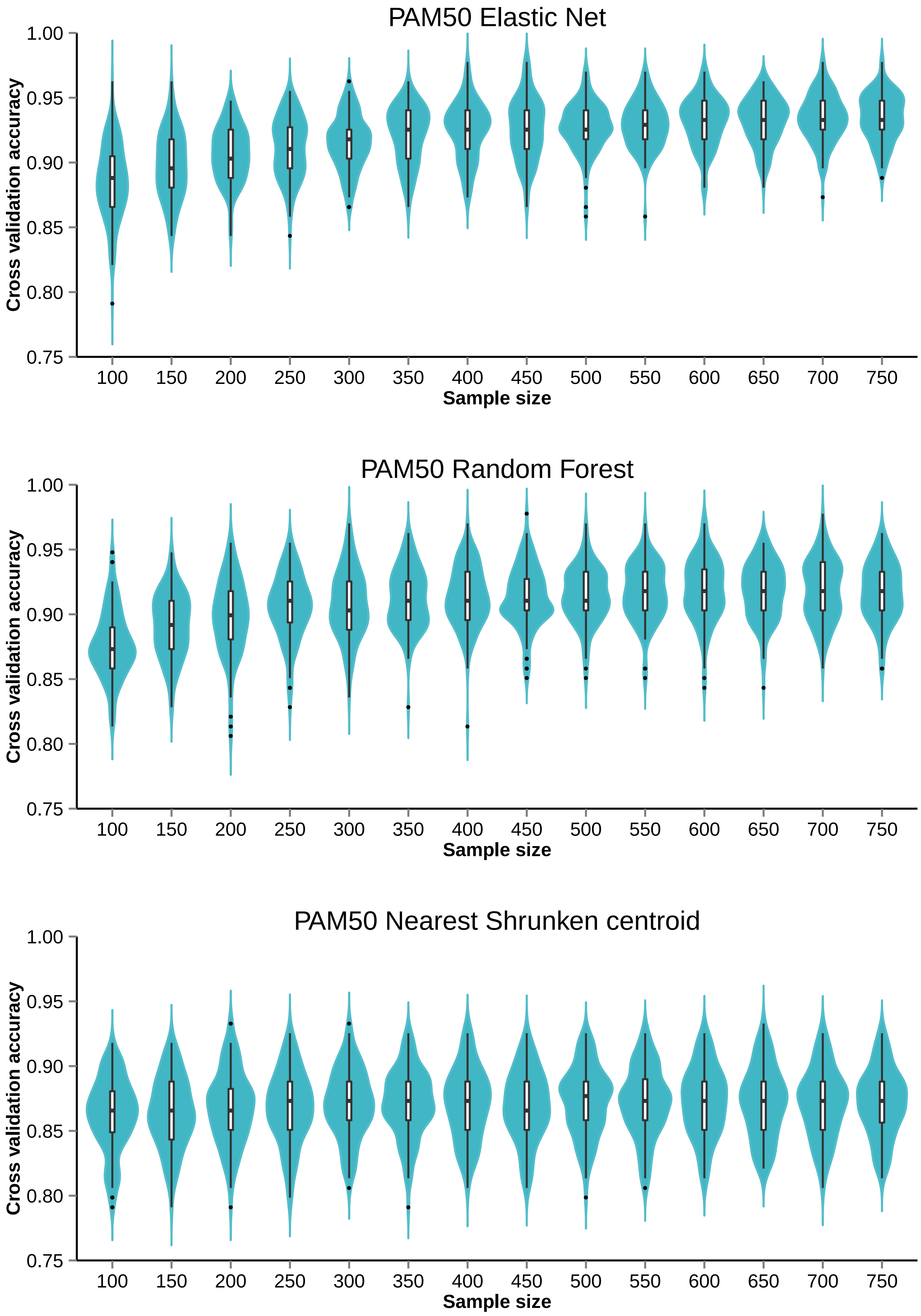

Figure S5: Violin plot visualisation of prediction accuracy over cross-validation rounds for PAM50 dataset at different training sample size

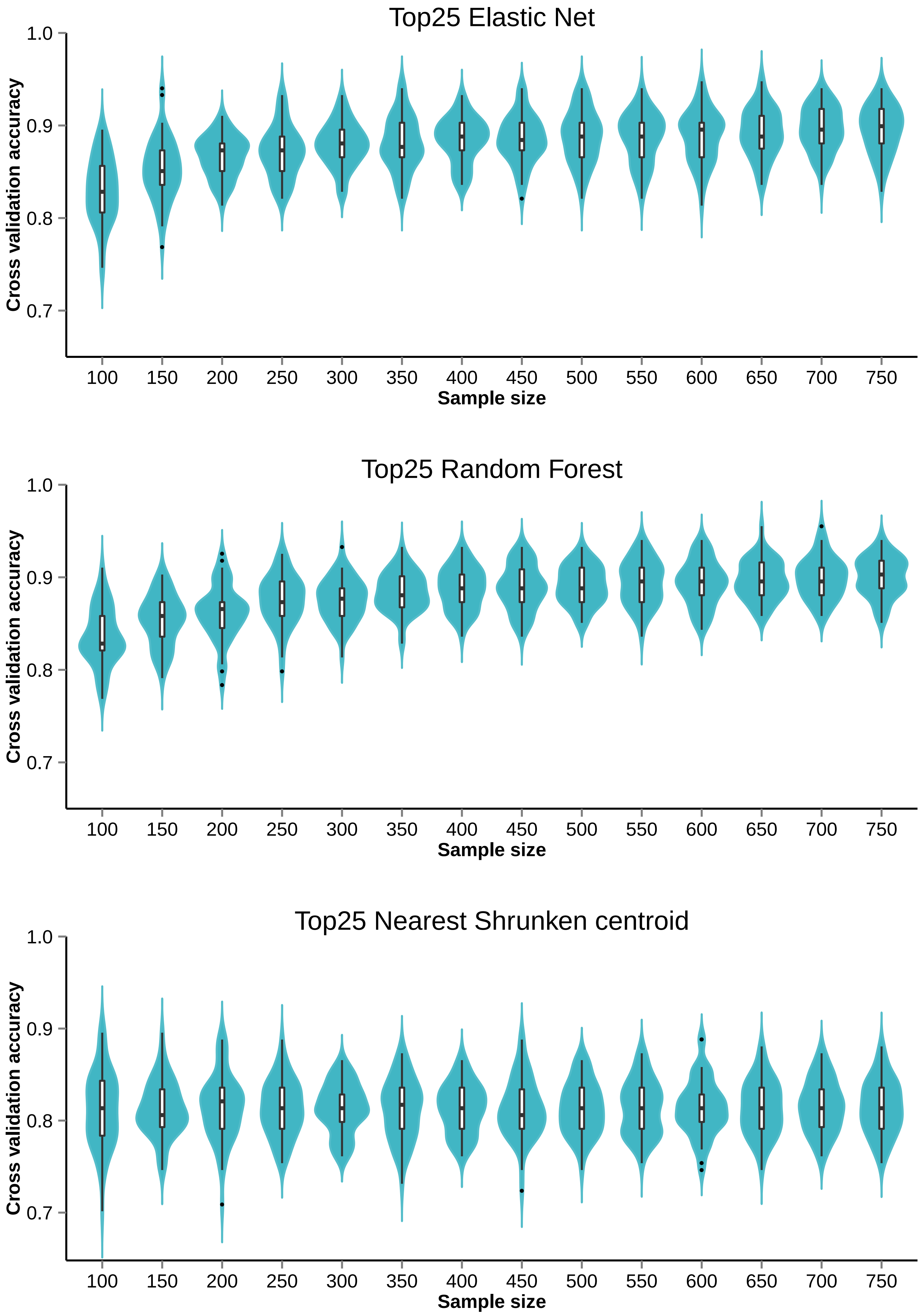

Figure S6: Violin plot visualisation of prediction accuracy over cross-validation rounds for Top25 dataset at different training sample size

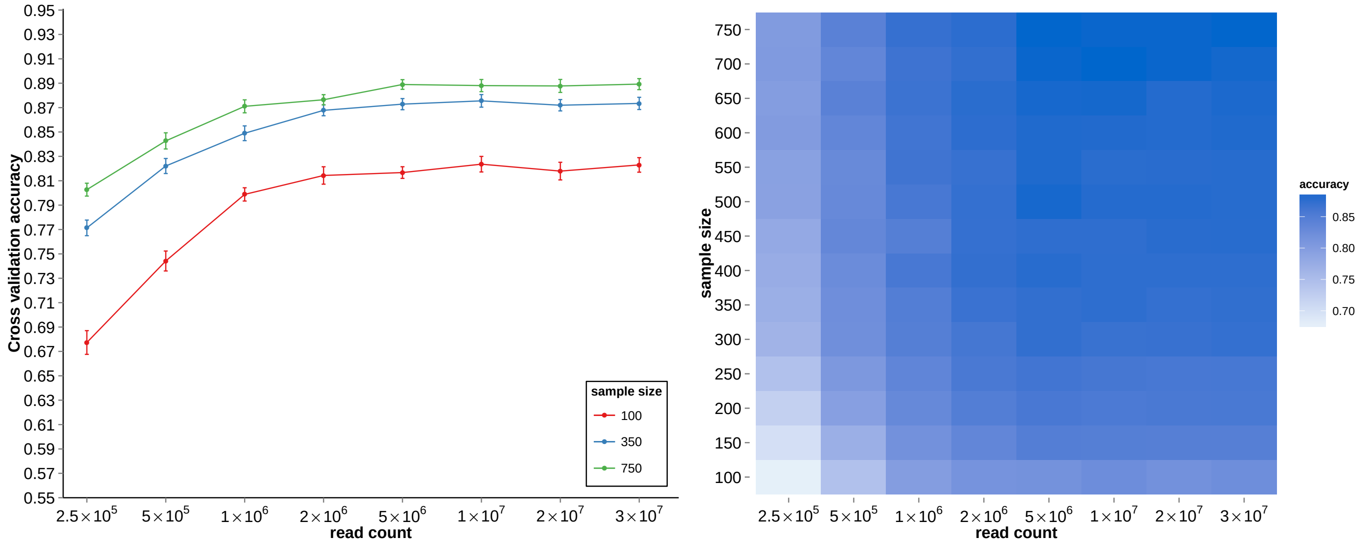

Figure S7: Effect of read count per sample on subtype prediction in Top25 dataset. (A) Classification accuracy at training sample size of 100, 350 and 750 (Error bars represent standard error of the cross-validation mean). (B) Heatmap representation of subtype classification accuracy and read count per sample

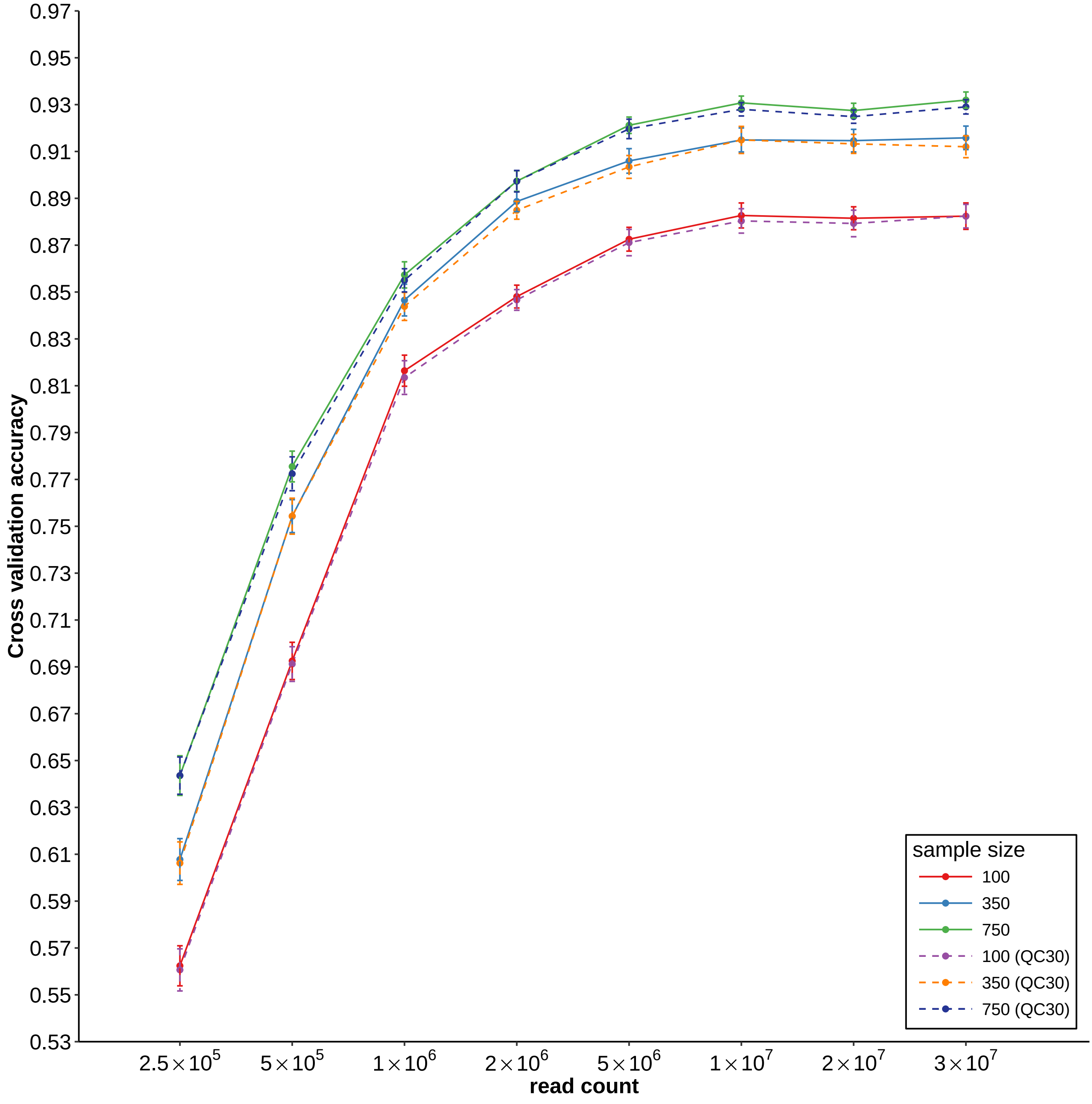

Figure S8: Effect of read count per sample on subtype prediction accuracy in PAM50 dataset, using htseq-count QC threshold 10 (solid lines) and QC threshold 30 (dashed lines). Classification accuracy at training sample size of 100, 350 and 750 (Error bars represent standard error of the cross-validation mean).

Table S1: Confusion matrix for PAM50 dataset and Elastic net classifier

sample size 100

True label \ Predicted label	Her2	Basal	LumA	LumB	Normal
Her2	0.876	0.014	0.023	0.086	0.001
Basal	0.028	0.964	0.000	0.005	0.004
LumA	0.013	0.000	0.880	0.084	0.023
LumB	0.035	0.000	0.134	0.831	0.000
Normal	0.021	0.015	0.046	0.007	0.911

sample size 150

True label \ Predicted label	Her2	Basal	LumA	LumB	Normal
Her2	0.898	0.013	0.014	0.075	0.000
Basal	0.024	0.968	0.000	0.003	0.006
LumA	0.012	0.000	0.880	0.084	0.024
LumB	0.032	0.000	0.098	0.870	0.000
Normal	0.021	0.008	0.034	0.009	0.927

sample size 200

True label \ Predicted label	Her2	Basal	LumA	LumB	Normal
Her2	0.912	0.010	0.015	0.063	0.000
Basal	0.027	0.965	0.000	0.002	0.006
LumA	0.011	0.000	0.894	0.071	0.025
LumB	0.028	0.000	0.093	0.879	0.000
Normal	0.020	0.009	0.036	0.006	0.929

sample size 250

True label \ Predicted label	Her2	Basal	LumA	LumB	Normal
Her2	0.925	0.007	0.010	0.058	0.000
Basal	0.025	0.967	0.000	0.003	0.005
LumA	0.011	0.000	0.895	0.070	0.024
LumB	0.030	0.000	0.077	0.893	0.000
Normal	0.020	0.011	0.037	0.006	0.927

sample size 300

True label \ Predicted label	Her2	Basal	LumA	LumB	Normal
Her2	0.914	0.010	0.014	0.061	0.001
Basal	0.024	0.971	0.000	0.001	0.004
LumA	0.008	0.000	0.902	0.067	0.023
LumB	0.027	0.000	0.076	0.897	0.000
Normal	0.019	0.011	0.034	0.005	0.931

sample size 350

True label \ Predicted label	Her2	Basal	LumA	LumB	Normal
Her2	0.945	0.007	0.009	0.038	0.001
Basal	0.022	0.974	0.000	0.002	0.003
LumA	0.008	0.000	0.902	0.067	0.023
LumB	0.023	0.000	0.065	0.912	0.000
Normal	0.019	0.009	0.034	0.006	0.932

sample size 400

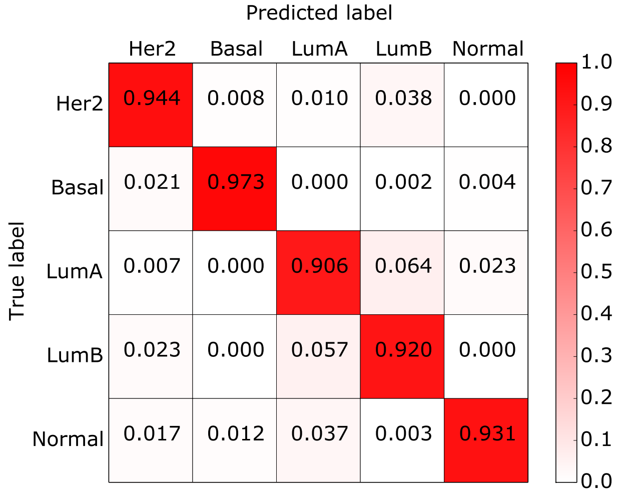

sample size 450

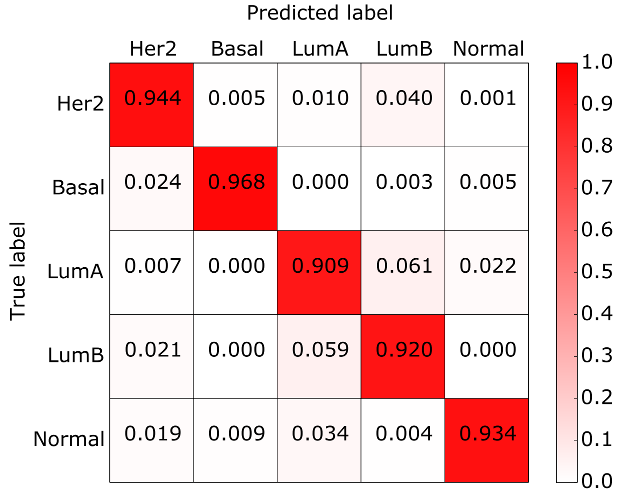

sample size 500

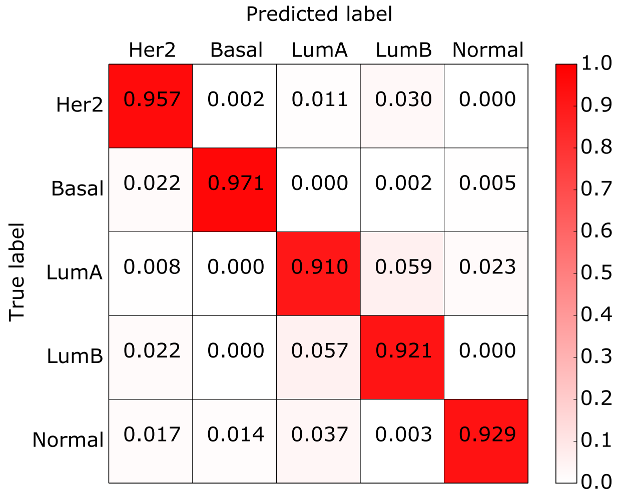

sample size 550

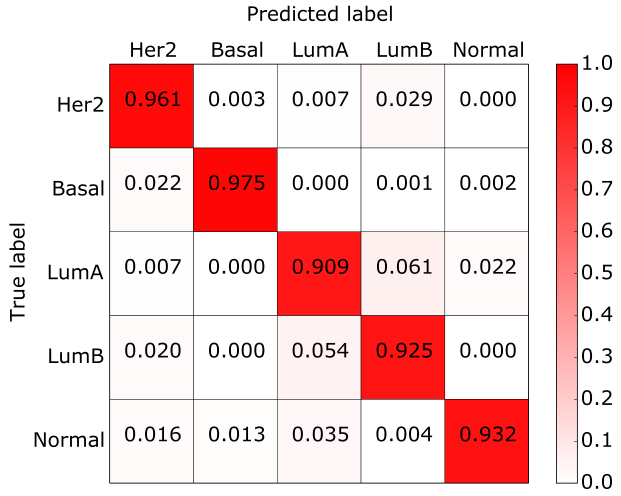

sample size 600

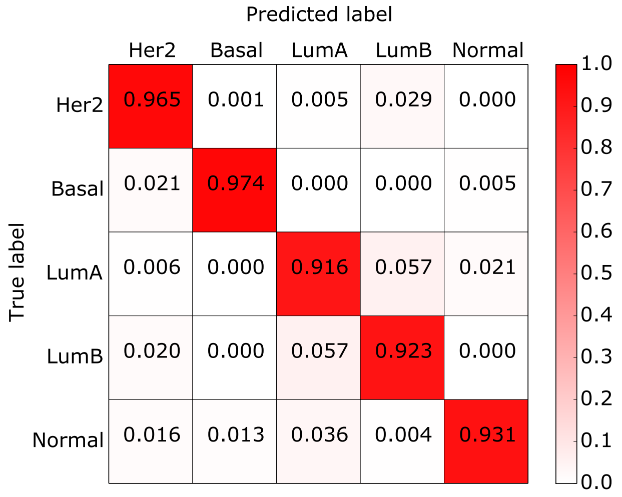

sample size 650

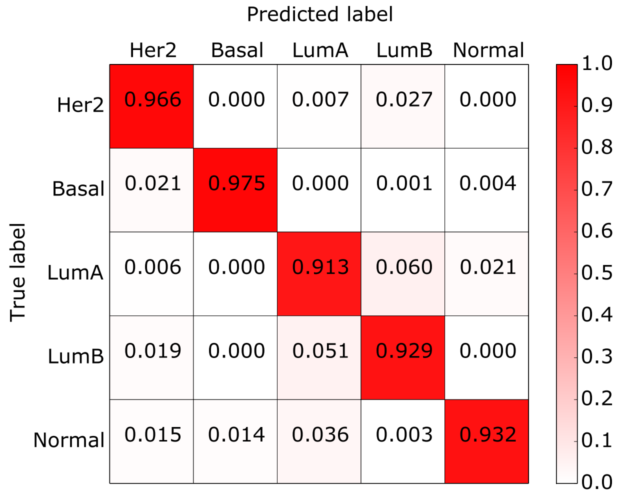

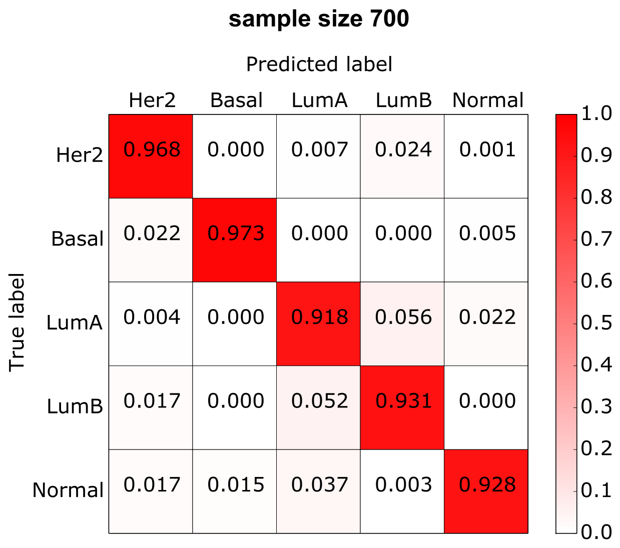
sample size 700
Predicted label
Her2 Basal LumA LumB Normal
True label
Her2 0.968 0.000 0.007 0.024 0.001
Basal 0.022 0.973 0.000 0.000 0.005
LumA 0.004 0.000 0.918 0.056 0.022
LumB 0.017 0.000 0.052 0.931 0.000
Normal 0.017 0.015 0.037 0.003 0.928
1.0 0.9 0.8 0.7 0.6 0.5 0.4 0.3 0.2 0.1 0.0

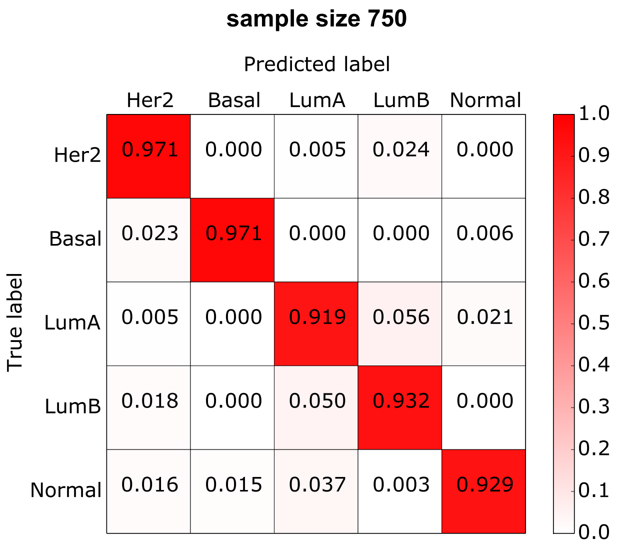
sample size 750
Predicted label
Her2 Basal LumA LumB Normal
True label
Her2 0.971 0.000 0.005 0.024 0.000
Basal 0.023 0.971 0.000 0.000 0.006
LumA 0.005 0.000 0.919 0.056 0.021
LumB 0.018 0.000 0.050 0.932 0.000
Normal 0.016 0.015 0.037 0.003 0.929
1.0 0.9 0.8 0.7 0.6 0.5 0.4 0.3 0.2 0.1 0.0

Table S2: Confusion matrix for Top25 dataset and Elastic net classifier

sample size 100

True label \ Predicted label	Her2	Basal	LumA	LumB	Normal
Her2	0.764	0.020	0.090	0.126	0.000
Basal	0.031	0.960	0.000	0.006	0.003
LumA	0.011	0.000	0.867	0.103	0.019
LumB	0.032	0.000	0.303	0.664	0.001
Normal	0.018	0.030	0.054	0.018	0.881

sample size 150

True label \ Predicted label	Her2	Basal	LumA	LumB	Normal
Her2	0.812	0.012	0.066	0.110	0.000
Basal	0.029	0.965	0.004	0.002	0.000
LumA	0.010	0.000	0.876	0.095	0.019
LumB	0.035	0.000	0.246	0.719	0.000
Normal	0.021	0.024	0.046	0.018	0.891

sample size 200

True label \ Predicted label	Her2	Basal	LumA	LumB	Normal
Her2	0.806	0.010	0.052	0.132	0.000
Basal	0.029	0.964	0.002	0.004	0.001
LumA	0.013	0.000	0.891	0.075	0.020
LumB	0.035	0.001	0.212	0.752	0.000
Normal	0.021	0.024	0.043	0.011	0.901

sample size 250

True label \ Predicted label	Her2	Basal	LumA	LumB	Normal
Her2	0.832	0.012	0.050	0.106	0.000
Basal	0.035	0.961	0.002	0.002	0.000
LumA	0.016	0.000	0.890	0.076	0.018
LumB	0.039	0.000	0.193	0.768	0.000
Normal	0.018	0.022	0.043	0.018	0.900

sample size 300

True label \ Predicted label	Her2	Basal	LumA	LumB	Normal
Her2	0.846	0.012	0.034	0.108	0.000
Basal	0.035	0.963	0.002	0.000	0.000
LumA	0.016	0.000	0.894	0.072	0.018
LumB	0.032	0.000	0.179	0.789	0.000
Normal	0.019	0.020	0.040	0.016	0.905

sample size 350

True label \ Predicted label	Her2	Basal	LumA	LumB	Normal
Her2	0.860	0.010	0.038	0.092	0.000
Basal	0.034	0.962	0.001	0.003	0.000
LumA	0.015	0.000	0.895	0.070	0.020
LumB	0.038	0.000	0.170	0.792	0.001
Normal	0.019	0.018	0.044	0.019	0.901

sample size 400

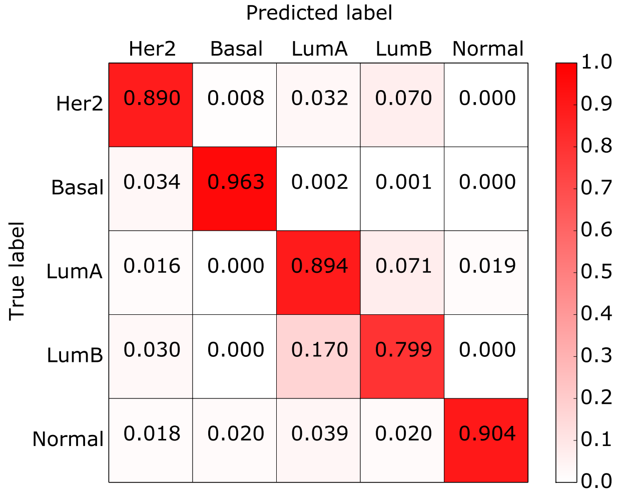

sample size 450

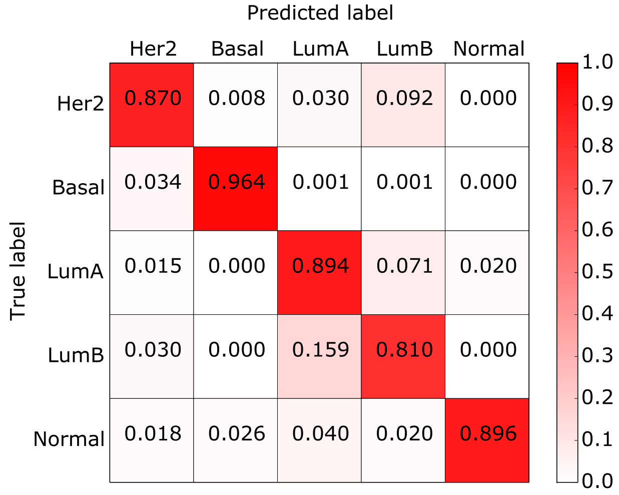

sample size 500

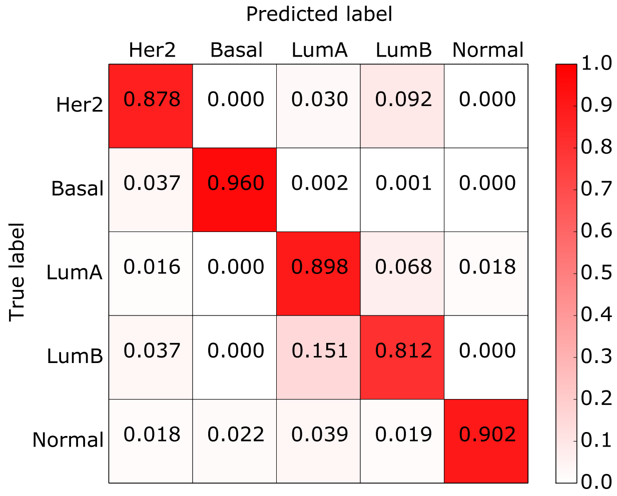

sample size 550

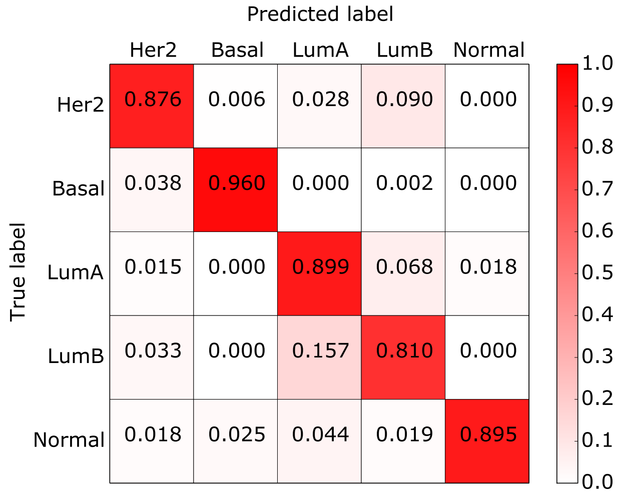

sample size 600

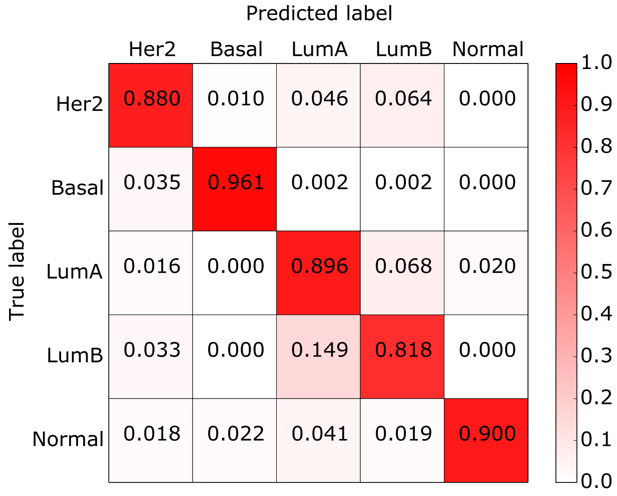

sample size 650

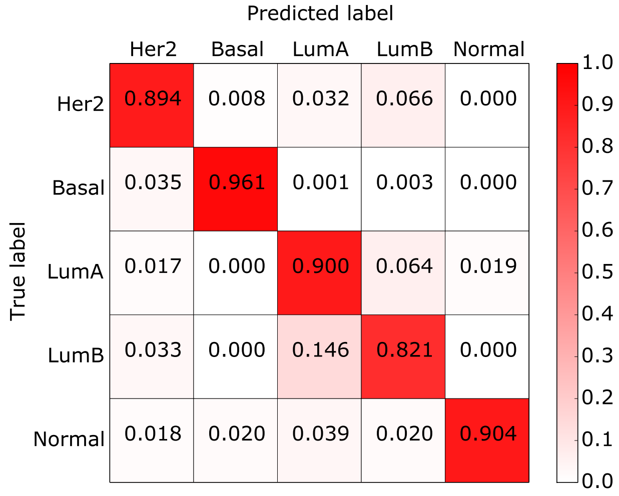

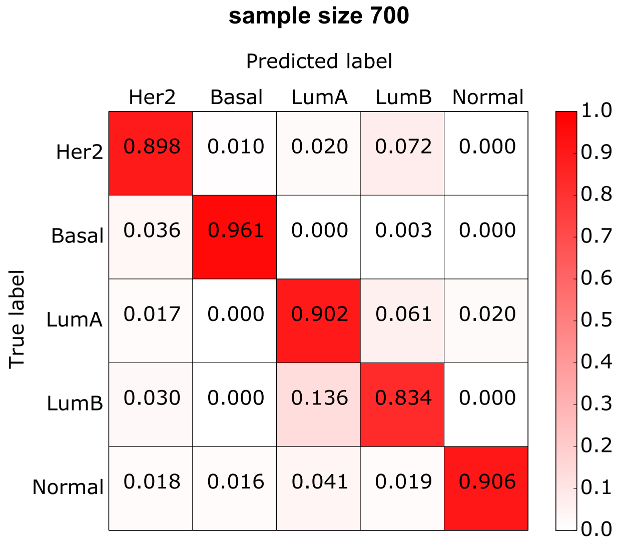
sample size 700
Predicted label
Her2
Basal
LumA
LumB
Normal
True label
0.898 0.010 0.020 0.072 0.000
0.036 0.961 0.000 0.003 0.000
0.017 0.000 0.902 0.061 0.020
0.030 0.000 0.136 0.834 0.000
0.018 0.016 0.041 0.019 0.906
1.0 0.9 0.8 0.7 0.6 0.5 0.4 0.3 0.2 0.1 0.0

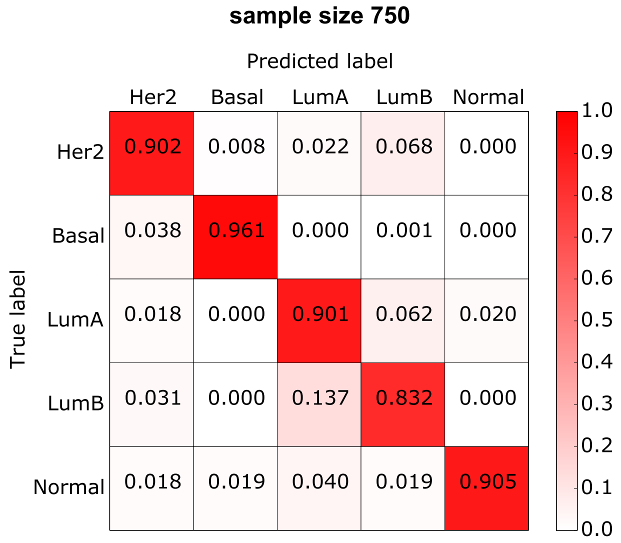
sample size 750
Predicted label
Her2
Basal
LumA
LumB
Normal
True label
0.902 0.008 0.022 0.068 0.000
0.038 0.961 0.000 0.001 0.000
0.018 0.000 0.901 0.062 0.020
0.031 0.000 0.137 0.832 0.000
0.018 0.019 0.040 0.019 0.905
1.0 0.9 0.8 0.7 0.6 0.5 0.4 0.3 0.2 0.1 0.0

Table S3: Confusion matrix for PAM50 dataset and unsupervised learning

sample size 100

True label \ Predicted label	Her2	Basal	LumA	LumB	Normal
Her2	0.888	0.044	0.000	0.068	0.000
Basal	0.007	0.989	0.000	0.004	0.000
LumA	0.031	0.000	0.652	0.166	0.151
LumB	0.123	0.000	0.174	0.703	0.000
Normal	0.017	0.044	0.005	0.020	0.915

sample size 150

True label \ Predicted label	Her2	Basal	LumA	LumB	Normal
Her2	0.873	0.049	0.000	0.078	0.000
Basal	0.006	0.990	0.000	0.005	0.000
LumA	0.030	0.000	0.661	0.160	0.149
LumB	0.115	0.001	0.180	0.704	0.000
Normal	0.017	0.046	0.005	0.019	0.912

sample size 200

True label \ Predicted label	Her2	Basal	LumA	LumB	Normal
Her2	0.883	0.047	0.000	0.071	0.000
Basal	0.005	0.990	0.000	0.005	0.000
LumA	0.031	0.000	0.667	0.166	0.137
LumB	0.108	0.000	0.155	0.737	0.000
Normal	0.018	0.044	0.003	0.019	0.916

sample size 250

True label \ Predicted label	Her2	Basal	LumA	LumB	Normal
Her2	0.892	0.047	0.000	0.061	0.000
Basal	0.004	0.991	0.000	0.005	0.000
LumA	0.031	0.000	0.670	0.166	0.134
LumB	0.111	0.000	0.140	0.750	0.000
Normal	0.018	0.043	0.003	0.020	0.916

sample size 300

True label \ Predicted label	Her2	Basal	LumA	LumB	Normal
Her2	0.900	0.052	0.000	0.048	0.000
Basal	0.005	0.990	0.000	0.005	0.000
LumA	0.032	0.000	0.654	0.180	0.134
LumB	0.110	0.000	0.113	0.776	0.000
Normal	0.018	0.045	0.004	0.019	0.914

sample size 350

True label \ Predicted label	Her2	Basal	LumA	LumB	Normal
Her2	0.896	0.044	0.000	0.060	0.000
Basal	0.004	0.992	0.000	0.004	0.000
LumA	0.032	0.000	0.663	0.173	0.133
LumB	0.111	0.000	0.135	0.753	0.000
Normal	0.018	0.044	0.002	0.020	0.916

sample size 400

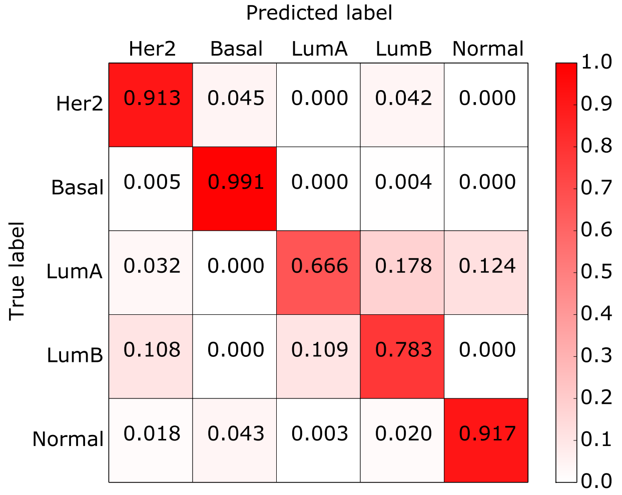

sample size 450

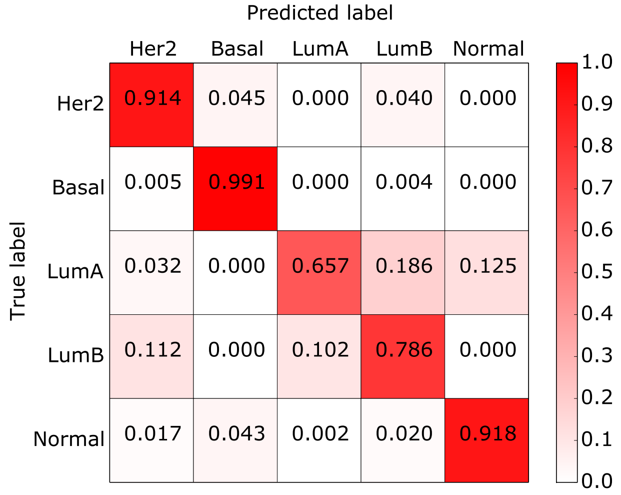

sample size 500

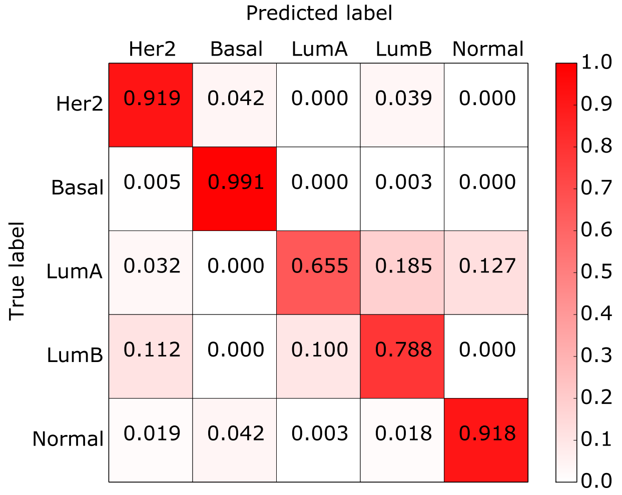

sample size 550

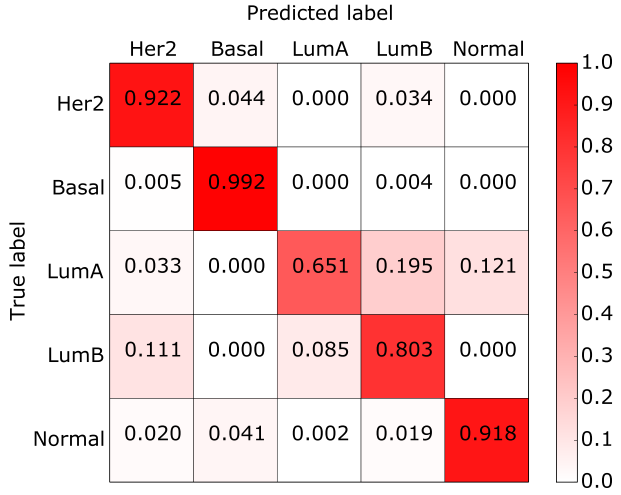

sample size 600

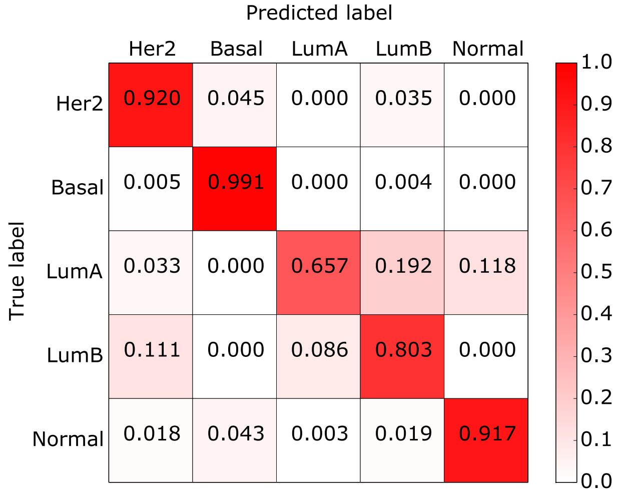

sample size 650

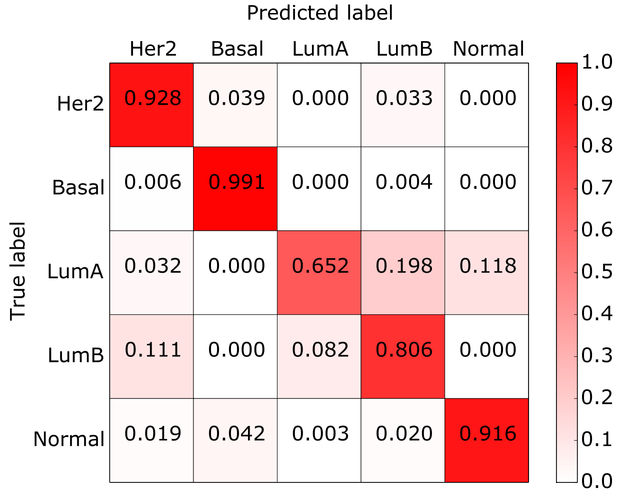

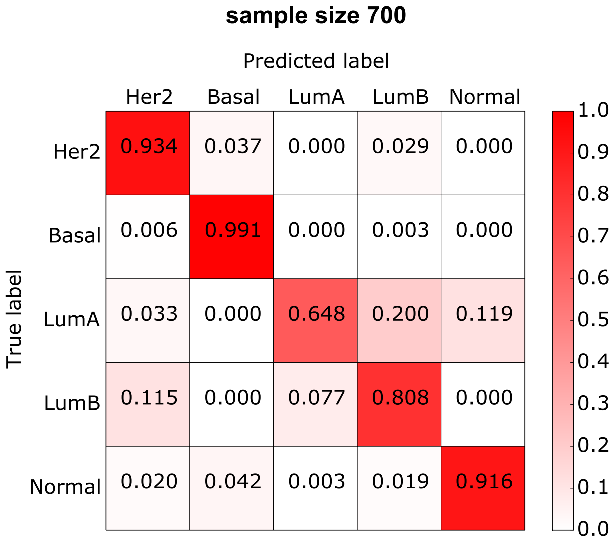
sample size 700
Predicted label
Her2
Basal
LumA
LumB
Normal
True label
Her2 0.934 0.037 0.000 0.029 0.000
Basal 0.006 0.991 0.000 0.003 0.000
LumA 0.033 0.000 0.648 0.200 0.119
LumB 0.115 0.000 0.077 0.808 0.000
Normal 0.020 0.042 0.003 0.019 0.916
1.0
0.9
0.8
0.7
0.6
0.5
0.4
0.3
0.2
0.1
0.0

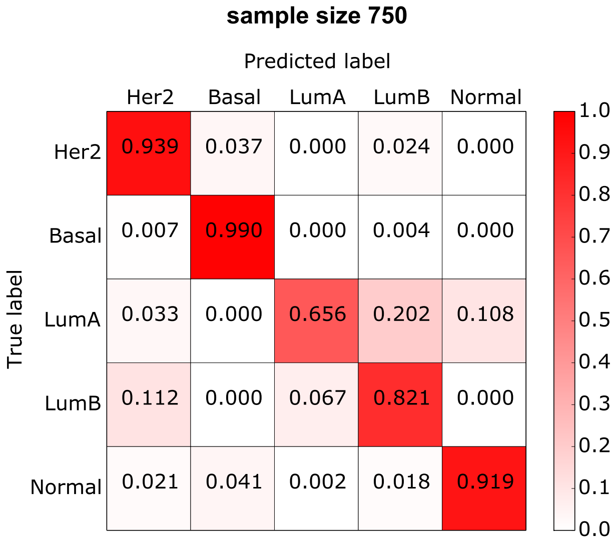
sample size 750
Predicted label
Her2
Basal
LumA
LumB
Normal
True label
Her2 0.939 0.037 0.000 0.024 0.000
Basal 0.007 0.990 0.000 0.004 0.000
LumA 0.033 0.000 0.656 0.202 0.108
LumB 0.112 0.000 0.067 0.821 0.000
Normal 0.021 0.041 0.002 0.018 0.919
1.0
0.9
0.8
0.7
0.6
0.5
0.4
0.3
0.2
0.1
0.0

Table S4: Confusion matrix for Top25 dataset and unsupervised learning

sample size 100

True label \ Predicted label	Her2	Basal	LumA	LumB	Normal
Her2	0.700	0.070	0.118	0.111	0.001
Basal	0.023	0.951	0.003	0.022	0.001
LumA	0.084	0.000	0.618	0.181	0.117
LumB	0.212	0.005	0.410	0.369	0.004
Normal	0.027	0.025	0.018	0.026	0.904

sample size 150

True label \ Predicted label	Her2	Basal	LumA	LumB	Normal
Her2	0.722	0.065	0.110	0.103	0.000
Basal	0.026	0.952	0.002	0.020	0.000
LumA	0.071	0.000	0.626	0.202	0.102
LumB	0.199	0.004	0.390	0.404	0.003
Normal	0.031	0.026	0.018	0.028	0.898

sample size 200

True label \ Predicted label	Her2	Basal	LumA	LumB	Normal
Her2	0.744	0.047	0.097	0.112	0.000
Basal	0.025	0.950	0.002	0.022	0.001
LumA	0.058	0.000	0.641	0.208	0.093
LumB	0.187	0.004	0.369	0.438	0.002
Normal	0.031	0.025	0.017	0.028	0.899

sample size 250

True label \ Predicted label	Her2	Basal	LumA	LumB	Normal
Her2	0.779	0.043	0.080	0.098	0.000
Basal	0.022	0.955	0.002	0.021	0.000
LumA	0.053	0.000	0.653	0.201	0.094
LumB	0.191	0.003	0.377	0.428	0.002
Normal	0.032	0.024	0.015	0.027	0.902

sample size 300

True label \ Predicted label	Her2	Basal	LumA	LumB	Normal
Her2	0.774	0.045	0.084	0.096	0.000
Basal	0.024	0.956	0.002	0.017	0.000
LumA	0.050	0.000	0.645	0.223	0.082
LumB	0.194	0.003	0.322	0.479	0.002
Normal	0.033	0.024	0.017	0.027	0.899

sample size 350

True label \ Predicted label	Her2	Basal	LumA	LumB	Normal
Her2	0.799	0.032	0.084	0.086	0.000
Basal	0.025	0.959	0.001	0.015	0.000
LumA	0.039	0.000	0.658	0.218	0.085
LumB	0.187	0.003	0.334	0.473	0.003
Normal	0.032	0.023	0.015	0.028	0.903

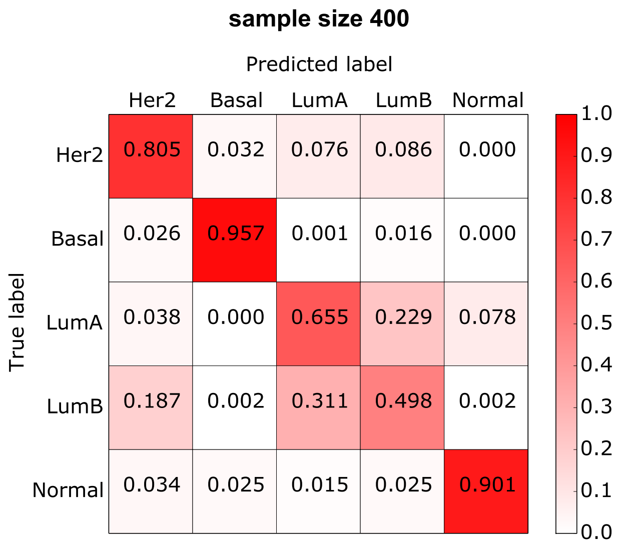
sample size 400
Predicted label
Her2 Basal LumA LumB Normal
True label
Her2 0.805 0.032 0.076 0.086 0.000
Basal 0.026 0.957 0.001 0.016 0.000
LumA 0.038 0.000 0.655 0.229 0.078
LumB 0.187 0.002 0.311 0.498 0.002
Normal 0.034 0.025 0.015 0.025 0.901
1.0 0.9 0.8 0.7 0.6 0.5 0.4 0.3 0.2 0.1 0.0

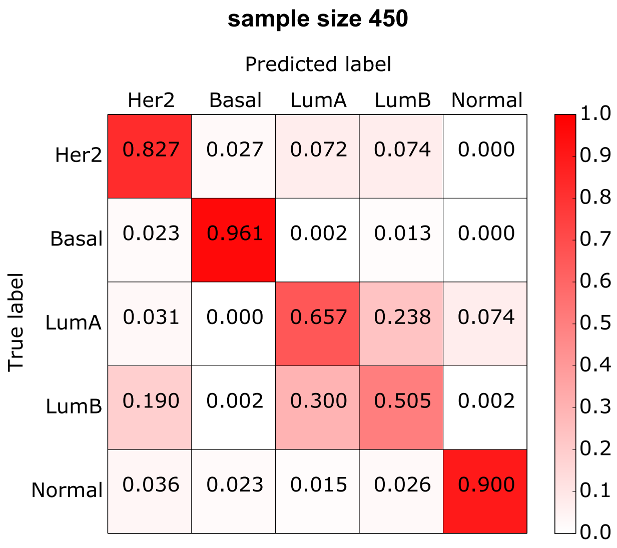
sample size 450
Predicted label
Her2 Basal LumA LumB Normal
True label
Her2 0.827 0.027 0.072 0.074 0.000
Basal 0.023 0.961 0.002 0.013 0.000
LumA 0.031 0.000 0.657 0.238 0.074
LumB 0.190 0.002 0.300 0.505 0.002
Normal 0.036 0.023 0.015 0.026 0.900
1.0 0.9 0.8 0.7 0.6 0.5 0.4 0.3 0.2 0.1 0.0

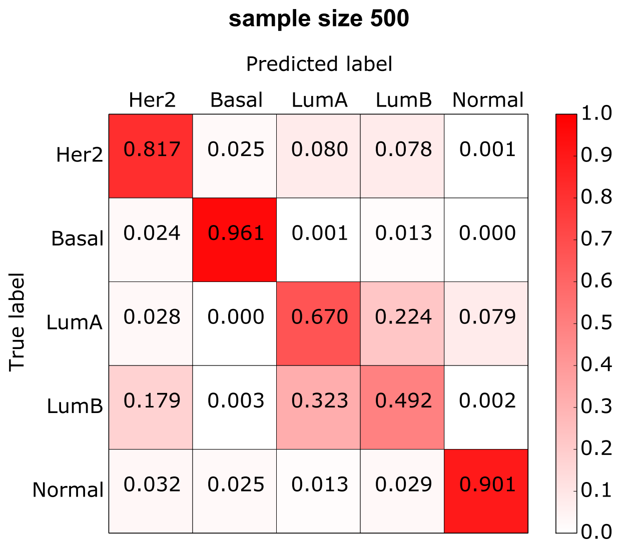
sample size 500
Predicted label
Her2 Basal LumA LumB Normal
True label
Her2 0.817 0.025 0.080 0.078 0.001
Basal 0.024 0.961 0.001 0.013 0.000
LumA 0.028 0.000 0.670 0.224 0.079
LumB 0.179 0.003 0.323 0.492 0.002
Normal 0.032 0.025 0.013 0.029 0.901
1.0 0.9 0.8 0.7 0.6 0.5 0.4 0.3 0.2 0.1 0.0

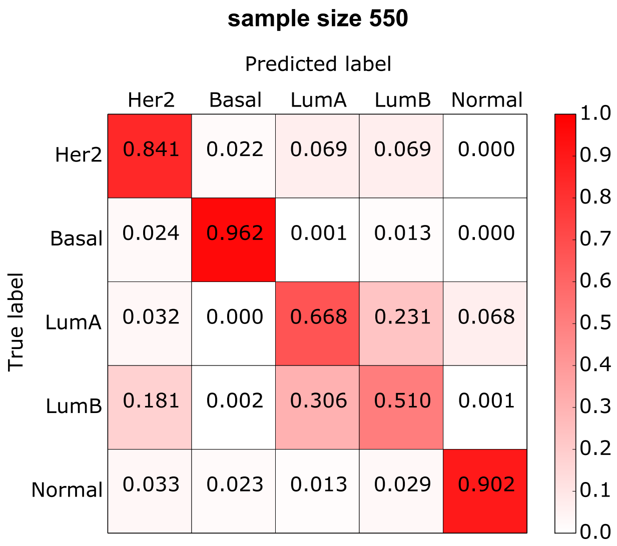
sample size 550
Predicted label
Her2 Basal LumA LumB Normal
True label
Her2 0.841 0.022 0.069 0.069 0.000
Basal 0.024 0.962 0.001 0.013 0.000
LumA 0.032 0.000 0.668 0.231 0.068
LumB 0.181 0.002 0.306 0.510 0.001
Normal 0.033 0.023 0.013 0.029 0.902
1.0 0.9 0.8 0.7 0.6 0.5 0.4 0.3 0.2 0.1 0.0

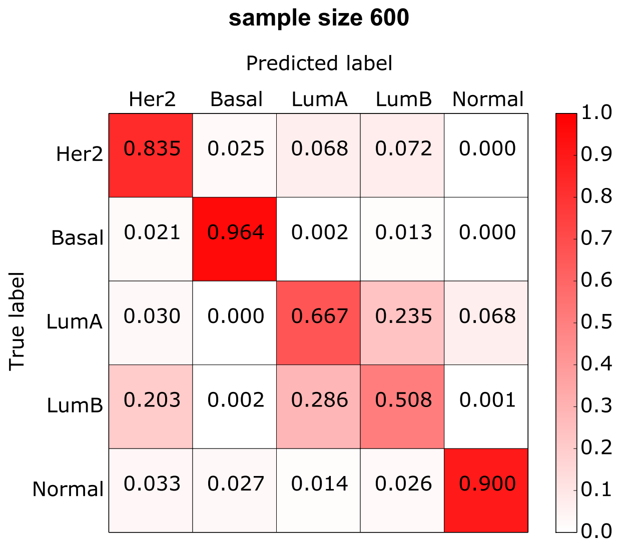
sample size 600
Predicted label
Her2 Basal LumA LumB Normal
True label
Her2 0.835 0.025 0.068 0.072 0.000
Basal 0.021 0.964 0.002 0.013 0.000
LumA 0.030 0.000 0.667 0.235 0.068
LumB 0.203 0.002 0.286 0.508 0.001
Normal 0.033 0.027 0.014 0.026 0.900
1.0 0.9 0.8 0.7 0.6 0.5 0.4 0.3 0.2 0.1 0.0

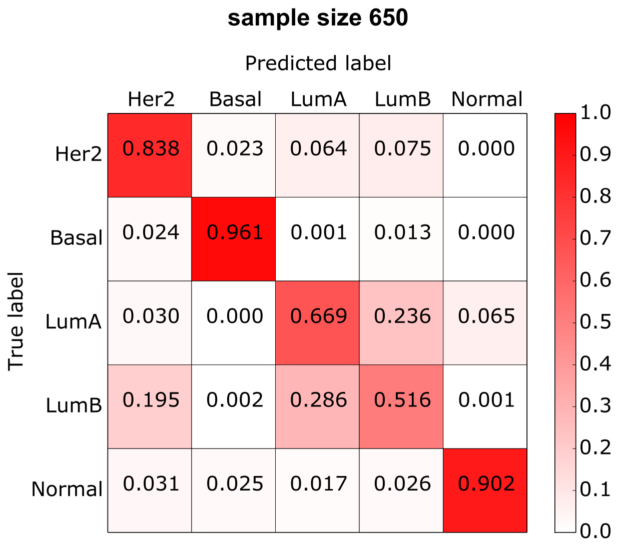
sample size 650
Predicted label
Her2 Basal LumA LumB Normal
True label
Her2 0.838 0.023 0.064 0.075 0.000
Basal 0.024 0.961 0.001 0.013 0.000
LumA 0.030 0.000 0.669 0.236 0.065
LumB 0.195 0.002 0.286 0.516 0.001
Normal 0.031 0.025 0.017 0.026 0.902
1.0 0.9 0.8 0.7 0.6 0.5 0.4 0.3 0.2 0.1 0.0

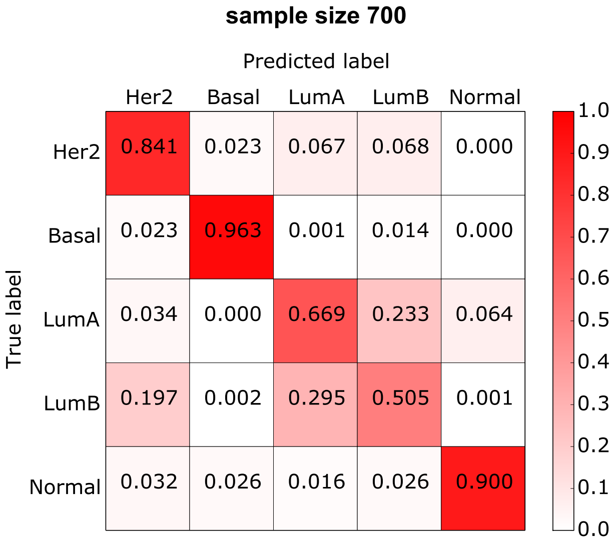
sample size 700
Predicted label
Her2 Basal LumA LumB Normal
True label
Her2 0.841 0.023 0.067 0.068 0.000
Basal 0.023 0.963 0.001 0.014 0.000
LumA 0.034 0.000 0.669 0.233 0.064
LumB 0.197 0.002 0.295 0.505 0.001
Normal 0.032 0.026 0.016 0.026 0.900
1.0 0.9 0.8 0.7 0.6 0.5 0.4 0.3 0.2 0.1 0.0

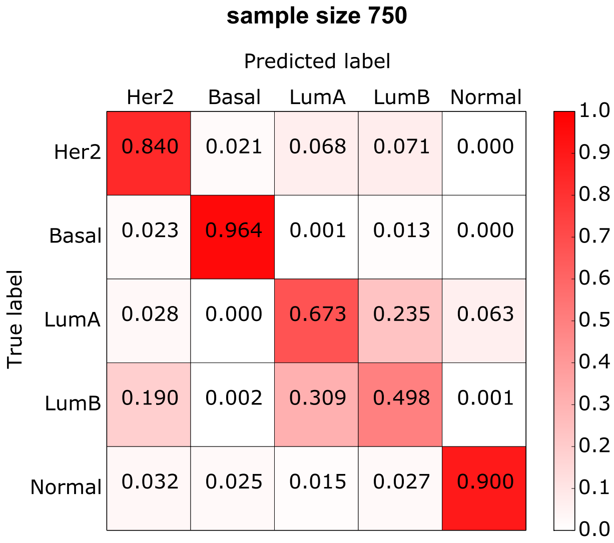
sample size 750
Predicted label
Her2 Basal LumA LumB Normal
True label
Her2 0.840 0.021 0.068 0.071 0.000
Basal 0.023 0.964 0.001 0.013 0.000
LumA 0.028 0.000 0.673 0.235 0.063
LumB 0.190 0.002 0.309 0.498 0.001
Normal 0.032 0.025 0.015 0.027 0.900
1.0 0.9 0.8 0.7 0.6 0.5 0.4 0.3 0.2 0.1 0.0
